# Epigenetic Regulation of MicroRNA Clusters and Families during Tumor Development

**DOI:** 10.3390/cancers13061333

**Published:** 2021-03-16

**Authors:** Jana Gregorova, Petra Vychytilova-Faltejskova, Sabina Sevcikova

**Affiliations:** 1 Babak Myeloma Group, Department of Pathophysiology, Faculty of Medicine, Masaryk University, 625 00 Brno, Czech Republic; j.gregorova@med.muni.cz; 2 Department of Molecular Medicine, Central European Institute of Technology (CEITEC), Masaryk University, 625 00 Brno, Czech Republic; petra.vychytilova@ceitec.muni.cz; 3 Department of Clinical Hematology, University Hospital Brno, 625 00 Brno, Czech Republic

**Keywords:** microRNA clusters, microRNA families, epigenetics, tumor development, DNA methylation, histone modifications, epigenetic therapy

## Abstract

**Simple Summary:**

In this review, the history of RNA interference discovery and current knowledge about microRNA biogenesis and post-transcriptional regulation of gene expression is summarized, with a special focus on microRNA clusters and families. Further, strong interplay between microRNAs and basic epigenetic mechanisms, such as DNA methylation and histone modifications, are introduced and associated with deregulated expression of microRNAs during tumor development. Finally, novel strategies for epigenetic-based therapies are discussed.

**Abstract:**

MicroRNAs are small non-coding single-stranded RNA molecules regulating gene expression on a post-transcriptional level based on the seed sequence similarity. They are frequently clustered; thus, they are either simultaneously transcribed into a single polycistronic transcript or they may be transcribed independently. Importantly, microRNA families that contain the same seed region and thus target related signaling proteins, may be localized in one or more clusters, which are in a close relationship. MicroRNAs are involved in basic physiological processes, and their deregulation is associated with the origin of various pathologies, including solid tumors or hematologic malignancies. Recently, the interplay between the expression of microRNA clusters and families and epigenetic machinery was described, indicating aberrant DNA methylation or histone modifications as major mechanisms responsible for microRNA deregulation during cancerogenesis. In this review, the most studied microRNA clusters and families affected by hyper- or hypomethylation as well as by histone modifications are presented with the focus on particular mechanisms. Finally, the diagnostic and prognostic potential of microRNA clusters and families is discussed together with technologies currently used for epigenetic-based cancer therapies.

## 1. History of microRNA Discovery

Twenty years ago, the classical dogma of molecular biology that DNA is transcribed into RNA, which is subsequently translated into proteins, was well accepted. However, with the development of whole-genome and transcriptome sequencing technologies, it was determined that more than 90% of the genome is actively transcribed. Surprisingly, less than 2% of the genome encodes proteins, while the rest of the transcriptome shows an extensive non-coding RNA (ncRNA) expression [1]. Previously, these molecules were considered to be “junk”, but it was found that most of them are involved in the majority of cellular processes and functions [2].

Today, microRNAs (miRNAs) belong to the well-documented small ncRNAs that are currently the subject of intensive translational research. The first described miRNA, *lin-4*, was discovered in *Caenorhabditis elegans* by Ambros et al. [3] and Ruvkun et al. [4] in 1993 as an essential regulator of control of developmental timing. In the December issue of Cell, both Ambros and Ruvkun reported that small and non-protein-coding transcript *lin*-4 regulates mRNA *lin-14* through its 3′ UTR region. Downregulation of LIN-14 protein was dependent on the transcription of *lin-4,* which is not translated into a protein [3,4]. However, it was only in 2000 when the second miRNA was identified by Ruvkun et al. [5]; it was *let-7*—a heterochronic gene of *C. elegans* which controls the transition of larval development through the binding to two closely spaced sites in *lin-41* 3′ UTR. Importantly, the *let-7* sequence is highly conserved across species from flies to humans [6]; the human let-7 family comprises 12 miRNAs that share complete sequence identity at the seed region, thus regulating the same targets [7]. These facts were significant for the study of miRNAs in other organisms.

Since then, thousands of miRNAs have been identified in humans and other species. According to the current version of miRBase database (version 22.1, October 2018; www.mirbase.org), 38,589 hairpin precursors and 48,860 mature miRNAs from 271 different organisms have been annotated. Concerning the human genome, 1917 hairpin precursors and 2654 mature sequences are included [8]. Besides miRBase, several other miRNA online databases, such as deepBase [9], microRNA.org [10], Rfam 14.4 [11], or miRGen v.3 [12], together with miRNA target prediction tools and software, including TargetScan [13], PicTar [14], TarBase [15], miRWalk [16], Diana-microT [17], or RNA22 [18], facilitate current studies investigating miRNAs involvement in cellular networks.

### 1.1. Biogenesis of microRNAs

Genes for miRNAs are distributed on all chromosomes. Interestingly, the lowest or no miRNA precursors are observed on the Y chromosome, while there are several chromosomes with extremely high numbers of miRNA genes in all species, probably due to clustering of miRNAs. In humans, chromosomes 1, 19, X, and 2 have the highest number of miRNA genes and constitute almost 30% of all miRNAs [19]. Previously, it was shown that miRNA genes are frequently located at fragile sites of chromosomes, in close to human papilloma virus integration sites, inside or near homeobox clusters, and in cancer-associated genomic regions commonly affected by deletions, amplifications, or breakpoints [20].

**Figure 1:**
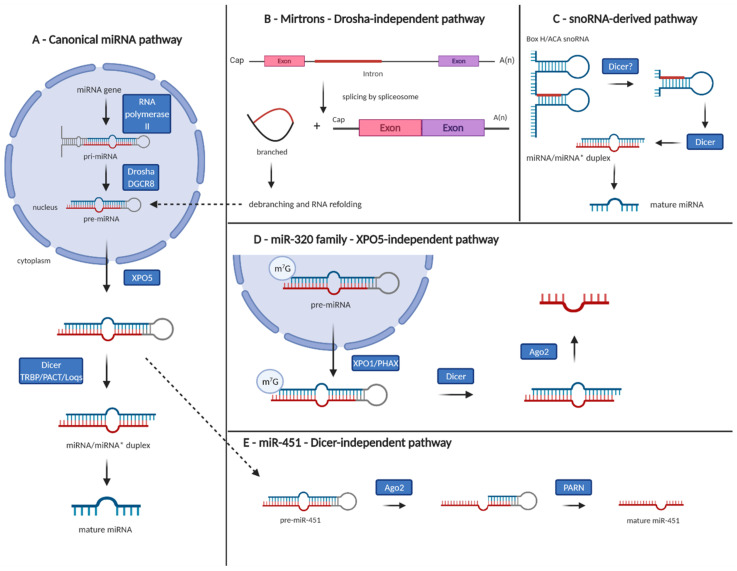
Canonical and non-canonical pathways of microRNA biogenesis. (**A**) Canonical pathway—microRNA gene is transcribed by RNA polymerase II into primary microRNA (pri-miRNA), cleaved by microprocessor complex Drosha/DGCR8, and precursor microRNA (pre-miRNA) is exported from the nucleus to the cytoplasm by Exportin 5 (XPO5) and further processed by Dicer and its partners into 18–25 nucleotide long microRNA duplex with 2-nucleotide 3′ overhangs. Guide strand is subsequently bound by the Argonaute proteins 1-4 (AGO1-4) and retained in the microRNA-induced silencing complex to target mRNAs for post-transcriptional silencing. (**B**) Mirtrons—generated through mRNA splicing independently of Drosha-mediated processing step. (**C**) Small nucleolar RNA-derived microRNAs—Drosha-independent pathway. (**D**) Exportin 5-independent transport of pre-miRNAs from the nucleus to the cytoplasm has been described in the case of miR-320 family. (**E**) Dicer-independent processing of miR-451—pre-miR-451 is directly loaded into AGO2, cleaved and trimmed by poly(A)-specific ribonuclease PARN to produce mature miR-451. The figure was created with BioRender.com.

Generally, miRNA genes may be divided into four subgroups: intergenic, intronic, exonic, and others (3′ UTR, 5′ UTR, and combinations of any two from intron, exon, 3′ UTR, and 5′ UTR) depending on their location in the genome [21]. It seems that while the intronic, exonic, and other miRNAs are the byproduct of transcription and post-transcriptional processing of corresponding host protein-coding genes, intergenic miRNAs have their own promoters and may be transcribed independently of protein-coding genes by RNA polymerase II [22]. They are transcribed as long primary transcripts (pri-miRNAs) that are capped and polyadenylated. The typical pri-miRNA consists of an imperfectly paired stem of about 33 base pairs (bp), with a terminal loop [23]. The pri-miRNA is subsequently recognized and cleaved by the so-called Microprocessor complex, which consists of the double-stranded RNase III enzyme Drosha and its essential cofactor, the double-stranded RNA-binding protein Di-George syndrome critical region 8 (DGCR8) [24]. This cleavage results in precursor miRNA (pre-miRNA) with a typical stem-loop structure, which is about 70 nt long. The pre-miRNA is exported to the cytoplasm by exportin 5 (XPO5)/RanGTP complex and further processed by Dicer (RNase III endonuclease). Dicer, together with its partners TRBP (HIV-1 trans-activating response RNA-binding protein) and PACT (protein kinase RNA activator), binds to the end of pre-miRNA and cuts both strands of the duplex, resulting in 18–25 nt long miRNA duplex with 2-nucleotide 3′ overhangs [25]. One of the strands (guide strand) is bound by the Argonaute proteins 1-4 (AGO1-4) and retained in the miRISC (miRNA-induced silencing complex) to guide the complex to complementary target mRNAs for post-transcriptional gene silencing. The second strand (passenger strand) is cleaved by AGO2 (Figure 1) [26].

While the biogenesis of most miRNAs is dependent on Drosha and Dicer, several non-canonical pathways have been described thanks to the analysis of next-generation sequencing (NGS) data. Non-canonical miRNAs have diverse origins, including introns, endogenous short hairpin RNAs (endo-shRNAs), endogenous short interfering RNAs (endo-siRNAs), tRNA precursors, and small nucleolar RNA (snoRNAs) (Figure 1) [27]. The Drosha-independent pathway is a non-canonical pathway where the Drosha-mediated processing step is bypassed. This pathway produces so-called mirtrons (short hairpin introns) that are generated through mRNA splicing; the entire process was firstly described in 2007 by Okamura et al. [28] in *Caenorhabditis elegans*, *Drosophila melanogaster*, and mammals. Other types of miRNAs produced by Drosha-independent pathway are miRNAs derived from snoRNAs. The snoRNAs are a conserved group of small non-coding RNAs; they associate with specific small nucleolar ribonucleoprotein (snoRNP proteins)—the complex that guides the enzymatic modification of selected ribosomal RNA (rRNA) nucleotides. Some snoRNAs are both the component of snoRNP and the source of miRNAs [29].

Another type of non-canonical pathway is the Dicer-independent pathway. Currently, there is only one known miRNA, which undergoes this pathway—vertebrate-specific miR-451 regulating erythroid development [30]. Biogenesis of this miRNA does not require Dicer and instead involves the catalytic activity of AGO2. Drosha cleaves pre-miR-451, which is loaded directly into AGO2; it is responsible for 3′-end trimming and maturation of pre-miR-451. Poly(A)-specific ribonuclease (PARN) trims down the 3′ end of AGO2-cleaved pre-miR-451 to produce mature miR-451 [31]. Finally, XPO5 independent transport of pre-miRNA from the nucleus to cytoplasm has been described in the case of miR-320 family, as these miRNAs use the complex of XPO1 and PHAX (phosphorylated adaptor for RNA export) proteins for their transport [32]. These results indicate high flexibility of the cells, enabling them to produce mature miRNAs from a broad spectrum of primary transcripts using diverse mechanisms independent of the key proteins of canonical biogenesis pathway (Figure 1).

### 1.2. Regulation of Gene Expression by microRNAs

The regulation of gene expression on post-transcriptional level was first described in 1998 by Andrew Z. Fire, Craig C. Mello, and their colleagues in *Caenorhabditis elegans*, when they proved the potential of short double-stranded RNAs (dsRNAs) to suppress mRNA translation based on sequence complementarity [33]. This process was termed RNA interference (RNAi); in 2006, both scientists were awarded the Nobel Prize in Physiology or Medicine for their discovery [34]. Today, several different short ncRNAs are known to be involved in RNAi processes, including miRNAs. These molecules are able to modulate the expression of 30–60% of protein-coding genes based on their complementarity to mRNA [35]. Importantly, a seed region represented by a conserved heptametrical sequence mostly situated at position 2–7 from the miRNA 5′ end is essential for this binding and commonly used for target predictions [36]. In addition, average miRNA has the potential to regulate approximately 100 target sites, while the expression of a particular gene may be affected by the binding of several different miRNAs [37]. Recent studies proved the potential of these molecules to bind not only the 3′ UTR regions but also the 5′ UTR regions [38] or coding sequences of target mRNAs [39]. Based on the degree of complementarity, the transcript undergoes degradation or its translation is silenced [40]. In 2010, Guo et al. [41] used ribosome profiling to measure the overall effects of miRNAs on protein production and mRNA levels. Their results showed that changes in mRNA levels closely reflected the impact of miRNAs on gene expression and indicated that destabilization of target mRNAs is the major mechanism responsible for reduced protein levels. Four years later, these results were confirmed by an independent study concluding that although translational repression is rapid, its effect is weak, while the effect of mRNA destabilization dominates [42]. Recently, Djuranovic et al. [43] proved that miRNA-mediated gene silencing happens during the initiation or early elongation phase of protein synthesis, and this inhibition is followed by mRNA deadenylation and decay. These findings could simplify the future of in vitro and in vivo studies analyzing the effects of miRNAs on mRNA and protein level changes.

In addition to mRNA repression, miRNAs have been also reported to activate gene expression, especially during cell starvation or other stress conditions. Vasudevan et al. [44] identified two proteins—AGO2 and FXR1 (Fragile X mental retardation gene 1)—activating the mRNA translation during cell starvation in vitro. They bind to AU-rich elements at 3′ UTR region of TNF-α (tumor necrosis factor α). Importantly, this binding is facilitated by miR-369-3. Similarly, let-7a and synthetic miRcxcr4 are able to activate the translation of specific mRNA in senescence cells, while the mRNA-miR-206 complex functions as a positive regulator of KLF4 (Krüppel-like factor 4) translation through its binding to translational control element (TCE) in RK3E epithelial cells [45]. In 2008, miR-373 was found to target the promoter of CDH1 (E-cadherin) and CSDC2 (cold-shock domain-containing protein C2), thus inducing their expression [46]. Two years later, decoy activity of miRNAs was described, when miR-328 was found to bind the hnRNP (heterogeneous ribonucleoprotein) E2 independently of the seed region and thus prevent its interaction with CEBPA (CCAAT enhancer-binding protein alpha) mRNA [47]. These findings reveal new mechanisms by which miRNAs may regulate gene expression. Nevertheless, post-transcriptional upregulation by miRNAs seems to be selective, specific to the RNA sequence context, cell type, and condition and associated with miRNP (miRNA ribonucleoprotein) factors or other RNA-binding proteins [48].

## 2. MicroRNA Clusters and Families

While protein-coding polycistronic transcripts in humans are rare, miRNAs are frequently clustered in genomes and transcribed as a single unit. Hence, multiple miRNAs may be produced from the same primary transcript, and their expression is regulated by the same factors, including epigenetic processes. Two or more miRNAs having close physical distance (less than 10 kb) are then called miRNA gene cluster [49]. Usually, two or three miRNAs are involved in a cluster, but larger clusters have also been described. According to the latest miRBase version (release 22.1, October 2018) [8], approximately 25% of human miRNAs (481 sequences) are located in 159 different clusters (GRCh38). The study of Guo et al. [49] from 2014 (135 clusters, 422 sequences) revealed that most miRNAs are prone to cluster on chromosomes 8, 17, and X, while the largest clusters may be found on chromosomes 19 (hsa-mir-512-1~1283-1 cluster, 46 members) and 14 (hsa-mir-379~656 cluster, 42 members). However, most of the clusters had 2–8 members, and 68% of them were composed of two miRNA genes (73% according to the newest data [8]). Interestingly, 19% of clusters were composed of multicopy miRNA genes. The multicopy pre-miRNAs could yield the same mature miRNAs, but they might be located in different genomic regions [49]. Importantly, clusters frequently contain representatives from different miRNA families; it has been proposed that mRNA transcripts coding for proteins that interact with each other are typically targeted by miRNAs from the same cluster [50]. As a large proportion of clustered miRNAs resides in close proximity to the fragile sites or cancer-associated genomic hotspots, their changed expression is commonly observed in various tumor types [51].

A miRNA gene family is defined as two or more miRNAs with high sequence similarity. It is supposed that the same family miRNAs are derived from identical ancestors in the phylogenetic tree [52]; at least a third of these families are highly conserved across species [53]. A miRNA family may be located in one or more clusters, and members of different families tend to be located in related clusters with close functional relationships [49]. In addition, miRNAs with a high degree of sequence homology differing in one or two nucleotides are annotated by adding a lowercase letter [54].

Currently, there are two major sources for miRNA family classification–miRBase [8] and Rfam database [11]. While miRBase classifies miRNAs into families based on sequence similarities in seed regions using BLAST [55] with manual adjustment, Rfam families are represented by multiple sequence alignment of known RNA sequences and a covariance model that can be used to search for additional family members [11]. Importantly, miRBase families have higher coverage but lower quality than Rfam miRNA families. In the current release, Rfam (14.4, December 2020) contains 526 human miRNA families [11], while miRBase (v.22.1, October 2018) annotated 2064 miRNA families [8]. Recently, several other classification approaches have emerged, including miRClassify [52], miRFam [56], or miROrtho [57]. Since mature miRNAs within one family share the same or very similar seed sequences, they may regulate nearly the same targets. It was also shown that miRNA families are involved in tumor development through the targeting of genes in cancer-related signaling pathways. Interestingly, Wuchty et al. [58] observed that miRNA families miR-27-3p, miR-181-5p, miR-124-3p.2/506-3p, and miR-200bc-3p/429 share similar pathway interaction patterns, whereas their seed sequences differ extensively. Importantly, as many predicted and literature-curated families are quite large, it is supposed that specific miRNA subsets with the seed sequence similarities rather than whole miRNA families contribute to the regulation of important signaling pathways associated with cancer development.

## 3. The Interconnection between microRNAs and Epigenetics

Epigenetics is the study of processes that alter gene activity without changing the DNA sequence; they may be heritable. Today, several different epigenetic processes are known, including DNA methylation, histone modifications, and regulation by ncRNAs, especially miRNAs.

DNA methylation is one of the most described epigenetic processes in tumor development, which usually occurs in the promoter region of genes at the cytosine bases, which are converted to 5-methylcytosine by different members of DNA methyltransferase (DNMT) enzymes. In mammals, methylation was observed to be globally distributed throughout the whole genome except for CpG islands, where high CpG contents are found. Importantly, inappropriate methylation of these sequences may lead to the silenced expression of key tumor suppressor genes in cancer cells [59]. Once established, DNA methylation patterns are maintained through cell divisions. However, recent studies suggest that ten-eleven translocation methylcytosine dioxygenases (TET1-TET3) may remove these methylation marks [60].

Concerning histones, post-translational modifications of amino-terminal tails including acetylation, methylation, phosphorylation, ADP ribosylation, or ubiquitination are commonly observed. Histone acetylation is catalyzed by histone acetyltransferases (HATs) that act as transcriptional co-activators. On the contrary, histone deacetylases (HDACs), which remove acetyl group from acetyl-lysin, function as transcriptional co-repressors [61]. Besides that, polycomb repressive complexes 1 and 2 (PRC1 and PRC2) have been identified in mammals to regulate gene expression. While the PRC2 trimethylates histone H3 on lysine 27 (H3K27me3) using Enhancer of Zeste homolog 2 (EZH2) catalytic subunit, PRC1 ubiquitylates histone H2A and compact polynucleosomes by RING1A/B and a Polycomb group ring finger protein, such as BMI-1 [62]. Interestingly, long non-coding RNAs (lncRNAs), such as XIST [63], HOTAIR [64], or ANRIL [65] have been found to be involved in PRCs recruitment and transcriptional repression. Finally, the SWI/SNF (SWItch/Sucrose Non-Fermentable) complexes that use the energy of ATP hydrolysis to remodel nucleosomes play an essential role in chromatin remodeling. Their catalytic activity is associated with BRG1 (SMARCA4) or BRM (SMARCA2) proteins; they have been shown to interact with other chromatin regulators, including HDACs [66] and PRCs [67]. Importantly, mutations in members of SWI/SNF complexes were detected in nearly 25% of human cancers [68].

Genes for miRNAs may be epigenetically regulated by DNA methylation and/or histone modifications what results in their overexpression or downregulation in numerous pathologies, including cancer. According to recent studies, almost half of miRNA genes are associated with CpG islands and almost 12% of them were prone to epigenetic inactivation by methylation in 23 different types of tumors. In addition, the long arms of chromosomes 1, 7, 11, 14, and 19 were found to be enriched by such genes [69]. Further, methylation was found to be involved in the regulation of expression of miRNA biogenesis genes [70], while multiple miRNAs can be also deregulated as a result of aberrant expression of specific epigenetic regulators, such as HDACs or PRCs. Scott et al. [71] proved that inhibition of HDACs results in transcription changes in 40% of miRNAs in SKBr3 cells. In turn, several miRNAs called epi-miRNAs were recognized to target enzymes involved in epigenetic modulation. It was observed that epigenetic regulators are strongly enriched among miRNAs predicted targets, indicating the importance of these small molecules in pluripotency, development, and cell reprogramming [72]. Recent studies also suggest that miRNAs may have direct epigenetic functions by recruiting specific protein complexes to genomic DNA [73]. These observations indicate the existence of a regulatory circuit between miRNAs and epigenetic modulation. Its disruption contributes to various diseases, including cancer.

## 4. Epigenetic Regulation of microRNA Clusters and Families during Tumor Development

**Figure 2:**
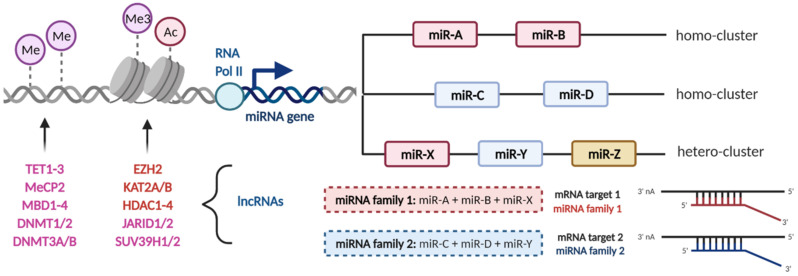
Epigenetic regulation of microRNA clusters and expression of families by DNA methylation and histone modifications. Aberrant DNA methylation and various histone modifications together with long non-coding RNAs (lncRNAs) are associated with deregulated microRNA expression in human cancers. The whole microRNA family may be located in the same cluster (homo-cluster) or in different clusters together with microRNAs from other families (hetero-cluster). MicroRNAs within one family share the same or very similar seed sequence, thus regulating the same targets. Me—methylation, Ac—acetylation, RNA Pol II—RNA polymerase II, TET1-3—ten-eleven translocation methylcytosine dioxygenases, MeCP2—methyl-CpG binding protein 2, MBD1-4—methyl-CpG-binding domain protein 1-4, DNMT—DNA methyl transferase, EZH2—enhancer of zeste homolog 2, KAT2A/B—histone acetyltransferases, HDAC1-4—histone deacetylases 1-4, JARID1/2—Jumonji/ARID1 B histone demethylase, SUV39H1/2—histone lysine N-methyltransferase. The figure was created with BioRender.com.

Global hypomethylation is a well-established epigenetic modification observed in various tumors. Researchers have reported hypomethylation of several important miRNA clusters, independently of non-specific global hypomethylation, associated with the re-activation of corresponding miRNAs [74,75,76]. On the contrary, site-specific DNA hypermethylation of promoter-associated CpG islands that leads to silencing of tumor-suppressive miRNA clusters is a hallmark of many human cancers [77]. Sometimes, the entire miRNA family is located in the same cluster, thus changed methylation pattern may result in deregulated expression of all target genes containing the corresponding seed region as miRNAs from such families. Thus, it is highly probable that aberrant DNA methylation is a major mechanism responsible for miRNA deregulation during tumor development. In addition, histone modifications have been previously described to be involved in epigenetic regulation of miRNAs expression (Figure 2) [78,79,80]. In the following paragraphs, the most studied miRNA clusters and families affected by hyper- or hypomethylation as well as by histone modifications are presented with the focus on particular mechanisms.

### 4.1. Let-7-5p/98-5p Family, miR-125-5p Family, miR-99-5p/100-5p Family

One of the largest miRNA families, let-7, including let-7a-g-5p, let-7i-5p, miR-98-5p, miR-4458, and miR-4500, is localized in six different clusters on chromosomes 9, 11, 19, 21, 22, and X in the human genome together with miR-99a, miR-99b, miR-100, miR-125a, miR-4763, and miR-10526. Interestingly, miR-99a, miR-99b, and miR-100 constitute another family as they contain the same seed region [8]. The first papers describing the role of DNA methylation in let-7 family expression were published in 2007. Lu et al. [81] found the association between the downregulated levels of tumor-suppressive let-7a-3 and its elevated methylation. This methylation further affected the expression of insulin-like growth factor-II (IGF-II) and the survival of ovarian cancer (OC) patients. Similarly, hypermethylation of promoter region of let-7a-3/let-7b cluster was confirmed in acute lymphoblastic leukemia (ALL) [82,83]. Using 5-aza-2’-deoxycytidine (5-Aza-CdR), the levels of let-7b increased significantly, inhibited proliferation of MOLT-4 cells and arrested the cells in G1 phase [83]. In contrast, hypomethylation of the same promoter was observed in lung adenocarcinomas [84] and myelodysplastic syndrome (MDS). Reactivation of the let-7a-3 expression was connected to oncogenic changes in lung transcription profiles [84] and shorter overall survival (OS) of MDS patients [85]. Concerning the let-7 family, let-7e is expressed from chromosome 19 in a cluster together with miR-99b and miR-125a. Hypermethylation of let-7e was detected in breast cancer (BC); decreased levels of this miRNA were associated with elevated cell proliferation, lower apoptosis, and poor prognosis [86]. Similarly, hypermethylation-associated silencing of miR-125a was observed in multiple myeloma (MM) [87], colorectal cancer (CRC) [88], or gastric cancer (GC) [89]. Interestingly, histone methyltransferase SUV39H1was identified as a target gene of this miRNA in GC; it was further confirmed that epigenetically silenced miR-125a-5p can be self-activated through targeting of this methyltransferase [89]. Finally, decreased levels of miR-98 in glioma tissues and cell lines were associated with increased DNA methylation and subsequently with a more aggressive tumor phenotype, increased invasion, and shorter survival of patients [90].

Concerning histone modifications, Mitra et al. [91] demonstrated epigenetic repression of let-7e in BC by binding of the histone demethylase Jumonji/ARID1 B (JARID1B) to its promoter region and removal of the H3K4me3 histone mark associated with active gene expression. Two years later, cytotoxin-associated gene A (CagA) of *Helicobacter pylori* (*H. pylori*) was identified to enhance the expression of c-myc, DNMT3B, enhancer of zeste homolog 2 (EZH2), and to attenuate the expression of miR-26a and miR-101, which resulted in the inhibition of let-7 expression through histone and DNA methylation in *H. pylori*-related gastritis and GC [92]. In lung cancer (LC), protein arginine methyltransferase 5 (PRMT5) was found to be overexpressed and to repress transcription of the miR-99 family by symmetrical dimethylation of histone H4R3, which increased fibroblast growth factor receptor 3 (FGFR3) expression, activated ERK1/2 and AKT, and in turn facilitated cell proliferation and metastases [93] (Table 1).

### 4.2. miR-34-5p/449-5p Family, miR-34b-5p/449c-5p Family

Although the sequence of seed region for miR-34s and miR-449s differs only in one nucleotide, these miRNAs are categorized into two different families—one consisting of miR-34a, miR-34c, miR-449a, and miR-449b, while the second one includes miR-34b, miR-449c, and miR-2682. Interestingly, miR-449a-c are localized together in one cluster on chromosome 5, while miR-34b and miR-34c are clustered on chromosome 11 [8]. MiR-34s are considered to be important tumor-suppressive miRNAs; they are the most studied miRNAs in various tumors concerning epigenetic regulation [96]. In 2008, inactivation of miR-34a by CpG methylation was described in prostate, breast, lung, colon, kidney, and pancreatic carcinoma cell lines; re-expression of this miRNA induced senescence and cell cycle arrest by targeting cyclin-dependent kinase 6 (CDK6) [97]. Two years later, Chim et al. [98] studied the role of methylation of this miRNA in a large panel of hematologic malignancies, including acute myeloid leukemia (AML), ALL, chronic lymphocytic leukemia (CLL), chronic myeloid leukemia (CML), MM, and non-Hodgkin’s lymphoma (NHL) using methylation-specific polymerase chain reaction (PCR). They found high methylation of miR-34a promoter in lymphoma (75%) and myeloma (37%) cell lines compared to normal controls and confirmed that hypomethylating treatment leads to re-expression of pri-miR-34a transcript in lymphoma cells with homozygous methylation. In primary samples at diagnosis, miR-34a methylation was detected in 4% of CLL, 5.5% of MM, and 18.8% of NHL samples, but no methylation was found in ALL, AML, and CML samples. Further, frequent concomitant inactivation of miR-34a and miR-34b/c by CpG methylation was observed in CRC, pancreatic cancer (PAC), BC, OC, urothelial carcinoma (UCA), and renal cell carcinoma (RCC). Interestingly, a statistically significant correlation of miR-34a methylation and the absence of p53 mutation in CRC was confirmed, suggesting that miR-34a inactivation may substitute for loss of p53 function in cancer [99]. To this day, the downregulation of miR-34s expression due to the elevated promoter methylation has been observed also in other tumors, including non-small cell lung carcinoma (NSCLC) [100], esophageal squamous cell carcinoma (ESSC) [101], malignant pleural mesothelioma (MPM) [102], HCC [103], laryngeal squamous cell carcinoma (LSCC) [104], bladder cancer (BLC) [105], or GC [106]. Importantly, 3,6-dihydroxyflavone was found to regulate the imbalance between DNA methylation and demethylation in BC by inhibiting DNMT1 activity and increasing TET1 expression. This effect led to demethylation of miR-34a promoter and increased levels of this tumor-suppressive miRNA [107]. Expression of miR-449a was found to be significantly silenced by DNA methylation in medulloblastoma (MB) [108] and NSCLC [109]. In addition, a negative correlation was observed between miR-449a levels and nicotinamide N-methyltransferase (NNMT) and knock-down of NNMT led to re-expression of miR-449a, inhibition of tumor growth, and activation of phosphatase and tensin homolog (PTEN) [109].

Concerning histone modifications, Lin et al. [110] proved that depletion of HDAC1 inhibits the metastatic abilities of GC cells by regulating the miR-34a/CD44 pathway. Similar results were observed also in cisplatin-resistant OC cell lines, where HDAC1 knockdown suppressed cell proliferation, increased apoptosis, and chemosensitivity by downregulating c-MYC and upregulating miR-34a [111]. Interestingly, expression of this miRNA may be negatively regulated also by lncRNA Linc-ROR that inhibits histone H3 acetylation in the miR-34a promoter [112]. Expression of miR-449a/b in tumors is epigenetically repressed through histone H3 Lys27 trimethylation; drug treatment targeting histone methylation resulted in strong induction of these miRNAs [113]. You et al. [114] indicated that this methylation is mediated through the zinc finger protein SUZ12, which is a part of the PRC2 complex. In HCC cells, upregulation of HDAC1-3 was detected and associated with the reduced levels of miR-449 [115]. Further, histone H3 lysine 27 acetylation (H3K27ac) was found to be altered during acquisition or resistance to anaplastic lymphoma kinase inhibitors in patients with ALK (anaplastic lymphoma receptor tyrosine kinase) fusion-positive LC; its decreased levels correlated with downregulation of tumor-suppressive miR-34a and miR-449a and increased proliferation [116] (Table 2).

### 4.3. The miR-141-3p/200a-3p Family, miR-200ab-5p Family, miR-200bc-3p/429 Family, miR-200c-5p/550a-3p Family

MiR-200s may be found in four different miRNA families together with miR-141-3p, miR-429, and miR-550a-3p. They are clustered and expressed as two separate polycistronic pri-miRNA transcripts—mir-200b-200a-429 located on chromosome 1 and mir-200c-141 located on chromosome 12 [8]. These miRNAs are frequently silenced in advanced cancers and have been implicated in epithelial-to-mesenchymal transition (EMT) and tumor invasion by targeting transcriptional repressors of E-cadherin ZEB1 and ZEB2. Interestingly, ZEB1 may repress mir-200c~141 cluster transcription in a negative feedback loop [120]. To this day, two different promoters of mir-200b~429 cluster with comparable activity and susceptibility to DNA methylation have been described [121]. In 2011, hypermethylation of miR-200s was detected and correlated with their decreased levels and poor prognosis of BLC patients [122]. In addition, resistance to cisplatin treatment was observed in patients with epigenetically silenced expression of these miRNAs [123]. On the contrary, hypomethylation and subsequent overexpression of miR-200a and miR-200b were found in PAC. In addition, both miRNAs were significantly increased in the sera of PAC and chronic pancreatitis patients compared to healthy donors, thus indicating clinical utility [124]. In the case of mir-200b~429 cluster, promoter methylation was confirmed in HCC, resulting in miR-200b downregulation, ZEB1 upregulation, and CD13 and CD24 expression. Interestingly, restoration of miR-200b expression was positively correlated with EpCAM. These results suggested that miR-200b-ZEB1 circuit may regulate diverse stemness of HCC [125]. On the other hand, mir-200b~429 cluster was found to be hypomethylated in pancreatic ductal adenocarcinoma (PDAC), while the expression of mir-200c~141 cluster was inhibited through promoter methylation. This downregulation was associated with the more invasive character and increased metastatic potential of PDAC cells in vitro and in vivo due to the enhanced expression of Wiskott-Aldrich syndrome protein-interacting protein family member 1 (WIPF1) and YAP/TAZ transcriptional co-activators [126].

Roy et al. [127] investigated tobacco-specific methylation patterns in miRNA loci associated with the prognosis of oral cancer patients. They found a significant correlation between hypermethylation of miR-200a/b and worse 5-year survival. Recently, flap endonuclease 1 (FEN1) was proven to interact with DNMT3A through proliferating cell nuclear antigen (PCNA) to suppress miR-200a-5p expression mediated by methylation, which resulted in increased levels of hepatocyte growth factor (MET) and epidermal growth factor receptor (EGFR) and thus elevating proliferation of BC cells [128]. Similarly, Kindlin 2 [129] and MYC protein [130] may form a complex with DNMT3A in the cell nucleus to induce CpG methylation of mir-200b~429 cluster promoter in BC. On contrary, DNA demethylase TET family members may activate transcription of epigenetically silenced miR-200s in BC and thus inhibit stemness, EMT, and metastasis formation [131]. Similarly, TET-dependent DNA demethylation was found to be essential for miR-200s, miR-141, and miR-429 reactivation and subsequent mesenchymal-to-epithelial transition in somatic cell reprogramming [132]. Interestingly, Choi et al. [133] suggested the direct involvement of *H. pylori* infection in epigenetic silencing of miR-200a/b through CpG methylation in gastric carcinogenesis. Finally, hypomethylation of miR-200a/b was found in local/local advanced prostate cancer (PC) patients, while hypermethylation was detected in patients with metastatic disease [117]. In addition, unmethylated promoter of mir-200c~141 cluster was found in LNCaP, 22RV1, and DU145 PC cell lines, while its hypermethylation was observed in PC3 cells [134]. These data indicate that epigenetic regulation of miRNAs expression through CpG promoter methylation is context-dependent.

The miR-200 family also controls the transition between cancer stem-cell-like and non-stem-cell-like phenotype of BC cells. While the mir-200c~141 cluster was silenced primarily by DNA methylation, mir-200b~429 cluster was repressed through polycomb group-mediated histone modifications [135]. Today, EZH2 is a well-known catalytic subunit of PRC2 complex responsible for H3K27 trimethylation. This enzyme is frequently upregulated in various human cancers, such as HCC [95], GC, or glioblastoma multiforme (GBM) [136] and is associated with epigenetic silencing of miRNAs expression, including miR-200 families. Importantly, EZH2 appeared to be essential for DNMT1 recruitment to the promoter region of mir-200b~429 cluster [136].

Recently, lncRNAs have been identified to play an important role in epigenetic regulation of miRNAs expression. The plasmacytoma variant translocation 1 gene (PVT1) and GIHCG have been reported to bind to EZH2 and recruit it to the mir-200b~429 cluster promoter in cervical cancer (CC) [137] and HCC [138]. Enkhbaatar et al. [139] observed that KDM5B, a histone H3K4 demethylase, represses the expression of the miR-200 family in cancer cells by changing the methylation status of its regulatory regions thus facilitating the EMT process and cancer progression. Concerning histone acetylation, proline, glutamic acid, and leucine-rich protein 1 (PELP1) were found to bind to miR-200a and miR-141 promoters and regulate their expression by recruiting chromatin modifier HDAC2 in BC [140]. In human lung adenocarcinoma cells, silencing of HDAC1/4 significantly increased miR-200b expression by upregulating H3 acetylation. As a result of miR-200b rescue, decreased levels of E2F3, survivin, and Aurora-A were detected, while the elevated levels of cleaved caspase-3 and higher sensitivity of cells to chemotherapy were observed [141]. Finally, P300 and PCAF (lysine acetyltransferase 2B—KAT2B) were identified as important co-factors of ZEB1 responsible for histone acetylation on the mir-200c~141 cluster promoter [142]. In addition, lncRNA H19 may form a complex with hnRNP U/PCAF/RNAPolII, activating miR-200 family expression in HCC by increasing histone acetylation [143]. These data suggest the development of combined anticancer therapy based on the targeting of miR-200 family together with lncRNAs (Table 3).

### 4.4. mir-17~92a-1 Cluster, mir-106a~363 Cluster

Mir-17~92a-1 is a polycistronic miRNA cluster located on chromosome 13; it includes six mature miRNAs: miR-17, miR-18a, miR-19a, miR-20a, miR-19b-1 and miR-92a-1. Cluster mir-106a~363 is found on chromosome X and contains miR-106a, miR-18b, miR-20b, miR-19b-2, miR-92a-2 and miR-363 [8]. Both clusters are commonly upregulated in various solid tumors and hematologic malignancies [144]. In addition, miR-17, miR-20a, miR-20b, and miR-106a belong to the same miRNA family based on the seed sequence similarity [8]. MiR-19a/b was found to directly promote multidrug resistance in GC cells. Demethylation of its promoter using 5-Aza-CdR led to increased expression of both miRNAs and subsequent downregulation of CpG binding protein 2 (MeCP2) via direct binding to its 3′-UTR [145]. Similar results were observed in the case of GBM, where the expression of miR-20a was negatively correlated to levels of DNMT1; overexpression of this miRNA was associated with resistance to temozolomide treatment. Subsequently, elevated levels of DNMT1 induced cell apoptosis [146]. Upregulated levels of miR-106a were observed in HCC [147] and GC [148] and inversely correlated with promoter methylation. Similarly, upregulated levels of miR-20b were detected in ESCC patients and associated with decreased promoter methylation, increased cell proliferation, migration, and invasion through inactivation of tumor-suppressive genes RB1 and TP53INP1 [149]. Decitabine treatment was performed to identify miRNAs influenced by methylation in NK/T-cell lymphoma (NKTL). Expression of miR-20b was epigenetically silenced in SNK6 cells, and STAT3 was detected as probable target of this miRNA [150]. In contrast, miRNAs from the mir-17~92a-1 cluster were identified as important tumor suppressors in PDAC; their expression was downregulated in tumor tissue via DNMT1 promoter hypermethylation [151,152]. Further, decreased levels of miR-18b were detected in melanoma by virtue of hypermethylation, and its expression was re-induced using 5-Aza-CdR [153]. Finally, lncRNA PVT1 was identified to promote gallbladder cancer proliferation by epigenetically suppressing miR-18b via DNA methylation [154].

The mir-17~92a-1 cluster was found to be aberrantly overexpressed in mixed-lineage leukemia-rearranged acute leukemias due to the elevated acetylation of histone H3 and H3K4 trimethylation. It plays an important role in the development of the disease through inhibiting cell differentiation and apoptosis while promoting cell proliferation [155]. Zhang et al. [156] demonstrated that acetylation of AGO2 specifically increases the binding of miR-19b into miRISC complex, thus enhancing its maturation. In addition, high levels of both miR-19b and acetylated AGO2 were associated with the poor prognosis of LC patients. HDAC inhibitor Vorinostat was shown to reduce levels of BRCA1-associated RING domain 1 (BARD1) by increasing the expression of miR-19a/b in AML [157] (Table 4).

### 4.5. miR-15-5p/16-5p/195-5p/424-5p/497-5p Family

The miR-15-5p/497-5p family consists of seven different miRNAs, including miR-15a-5p, miR-15b-5p, miR-16-5p, miR-195-5p, miR-424-5p, miR-497-5p, and miR-6838-5p. While miR-15a is clustered together with miR-16-1 on chromosome 13, miR-15b is located in another cluster on chromosome 3 together with miR-16-2, whereas mir-497~195 cluster is found on chromosome 17. Finally, miR-424 is clustered together with other miRNAs (miR-503, miR-542, miR-450a/b) from different families on chromosome X [8]. MiR-15a/b and miR-16 are well-known important tumor suppressors with downregulated levels in various hematologic malignancies. Hypermethylation of these two clusters was also associated with the progression of MDS into AML and poor prognosis [158]. Concerning the mir-497~195 cluster, its methylated promoter was identified in BC. Forced expression of these two miRNAs resulted in decreased proliferation and invasion of cells by targeting RAF-1, CCND1 [159], and mucin-1 (MUC1) [160]. Similar results were obtained in the case of miR-497 and GC cells [161]. Downregulation of this cluster was detected also in HCC. Interestingly, decreased expression of these miRNAs was affected not only by promoter hypermethylation but also by aberrant methylation status of their transcription factors NEUROG2 (neurogenin-2) and DDIT3 (DNA damage-inducible transcript 3) [162]. Significant downregulation of miR-195 due to its methylation was described also in PC. Using 5-Aza-CdR, levels of this miRNA increased, which resulted in suppressed cell proliferation, migration, invasion, and EMT of PC cells [163]. MiR-424 also functions as an important tumor suppressor, and its expression is inversely correlated with promoter DNA methylation in GBM [164], CC [118], endometrial endometrioid adenocarcinoma [165], and OC [166].

In 2012, mir-15a~16-1 transcriptional repression by c-Myc and HDAC3 in mantle cell and other non-Hodgkin B-cell lymphomas was described [167]. Later on, the same results were observed in CLL [79] and NSCLC; using HDAC inhibitor trichostatin A, levels of miR-15a and miR-16 increased significantly together with its host gene *DLEU2* [168]. HDAC3 can anchor to the miR-195 promoter via SP1 interaction and consequently can repress miR-195 transactivation by deacetylating histone in HCC cells [169]. Finally, lncRNA PVT1 directly interacts with EZH2, and the complex binds to the promoter region of miR-195, resulting in increased H3K27me3 levels, decreased expression of miR-195, and changed response to paclitaxel treatment in CC cells. Interestingly, direct sponging of miR-195 by PVT1 was also observed [170] (Table 5).

### 4.6. miR-23-3p Family, mir-23b~24-1 Cluster, mir-23a~24-2 Cluster

MiR-23-3p family includes four miRNAs: miR-23a-3p, miR-23b-3p, miR-23c, and miR-130a-5p. In addition, miR-23a is found in a cluster on chromosome 19 together with miR-27a and miR-24-2, while miR-23b is localized on chromosome 9 together with miR-27b, miR-24-1, and miR-3074 [8]. MiR-23a/b function as important tumor suppressors, and their downregulation may be caused by hypermethylation in numerous cancers, including osteosarcoma (OSS) [171], laryngeal cancer [172], PC [173], CC [174], MM, Waldenström macroglobulinemia [175], and HCC [176]. On the other hand, miR-23a elevation associated with CpG hypomethylation was observed in another study dealing with HCC patients [162]. Interestingly, miR-23b is epigenetically inactivated through its host gene C9orf3; this inactivation was connected to HPV-16 E6 infection in CC [177]. MiR-27a/b are dual-function miRNAs, meaning they can function as both oncogenes and tumor-suppressors based on the cellular context. MiR-27a was found to be epigenetically silenced by methylation in PC [178], while miR-27b hypermethylation was observed in tamoxifen-resistant BC. Importantly, re-expression of this miRNA after treatment with 5-Aza-CdR sensitized MCF-7 cells to tamoxifen, reversed EMT-like properties, inhibited invasion, and downregulated its target gene HMGB3 [179]. Similarly, miR-27b-3p was confirmed to be downregulated in GC, and treatment with 5-Aza-CdR enabled partial demethylation of CpG island in its promoter region [180]. Contrary to these results, miR-27b oncogenic properties were observed in CC as increased levels were associated with cell proliferation and invasion and reduced apoptosis. In addition, lncRNA TOB1-AS1 was able to degrade its expression. However, epigenetic silencing of TOB1-AS1 by promoter methylation restrained miR-27b inhibition and contributed to CC progression [181]. Downregulation of miR-24 associated with promoter hypermethylation was observed in PC [182]; however, the exact mechanism has not been described.

Recently, HDAC3 inhibition by class I-specific HDAC inhibitor entinostat was described to decrease the activity of the chromatin remodeling enzyme SMARCA4, which in turn de-repressed miR-27a. Elevated levels of this miRNA led to PAX3:FOXO1 mRNA destabilization and sensitization to chemotherapy in rhabdomyosarcoma cells [183]. Similarly, inhibition of HDAC6 in diffuse large B-cell lymphoma resulted in increased miR-27b levels, inhibition of MET signaling pathway, and decreased tumor growth [184] (Table 6).

### 4.7. miR-130-3p/301-3p/454-3p Family

Hypermethylation of miR-130-3p was described in PC tissues as well as in drug-resistant cell lines; its expression correlated with the level of methylation. Importantly, colony-stimulating factor 1 (CSF-1) was confirmed as a direct target of miR-130-3p responsible for decreased sensitivity to anticancer drugs. Thus, demethylation with 5-Aza-CdR led to reactivation of miRNA expression, downregulation of CSF-1, and decreased multidrug resistance [185]. Interestingly, miR-130b belongs to the same cluster as miR-301b, which is located on chromosome 22; these two miRNAs share target genes. Downregulation of this cluster mediated by elevated promoter methylation was detected in PC and associated with cell proliferation [186]. On contrary, Fort et al. [187] observed upregulation of these miRNAs in neoplastic and metastatic prostate tissue and described oncogenic role of this cluster. However, elevated levels were not due to decrease in DNA methylation, as the promoter of these genes was found to be lowly methylated in normal and neoplastic tissue. Concerning miR-454-3p, Bao et al. [188] analyzed the expression of this miRNA in chondrosarcoma tissues. They found downregulated levels of miR-454-3p and upregulated levels of lncRNA HOTAIR. Subsequently, they observed that HOTAIR is able to induce methylation of miR-454-3p by recruiting EZH2 and DNMT1 to promoter regions, which resulted in lower apoptosis and increased proliferation of cells. Thus, HOTAIR could serve as a promising therapeutic target for chondrosarcoma. Deregulated expression of miR-130a/b was observed also in endometrial cancer. Detailed analyses proved that 5-Aza-CdR and HDAC inhibitors may increase the levels of these miRNAs and inhibit the malignant behavior of cells [189] (Table 7).

### 4.8. miR-29-3p Family

The miR-29-3p family consists of three miRNAs: miR-29a-3p, miR-29b-3p, and miR-29c-3p that are clustered together on two different chromosomes, chromosome 1 (mir-29b-2~29c cluster) and chromosome 7 (mir-29b-1~29a cluster) [8]. These miRNAs are usually downregulated in various cancers, thus serving as tumor suppressors and important epi-miRNAs. However, only little is known about the epigenetic regulation of their expression. Mazzoccoli et al. [190] identified methylation of CpG sequences in promoter regions of both clusters; this methylation was decreased after decitabine treatment or DNMT3B inhibition by siRNA in Burkitt lymphoma cells. Interestingly, it was suggested that the expression of mir-29b-1~29a cluster may be suppressed by DNMT3B in a DNA-methylation-dependent manner, and in turn, miR-29a/b may suppress DNMT3B by binding to its 3‘-UTR region. Deregulation of miRNAs as well as DNMT3A led to the epigenetic silencing of CDH1 and contributed to the metastasis formation in GC [191]. In addition, LASP1 was confirmed as a direct target of epigenetically silenced miR-29b responsible for invasive potential of GC cells [192]. These results were subsequently confirmed also in OC [193], and similar data were achieved for DNMT1 and miR-29b in PAC [194]. Interestingly, the expression of this miRNA may be downregulated through methylation by lncRNA DCST-AS1 in GBM [195].

Epigenetic regulation of miR-29 family through histone modifications was firstly described in 2010 when SP1 was found to participate in a NFkappaB/HDAC complex that repressed miR-29b transcription in AML [196]. Similarly, PRMT5 is able to interact with SP1 in a transcription repressor complex and silence miR-29b via histone 4 arginine residue H4R3 dimethylation, which results in transcription activation of FLT3 receptor tyrosine kinase [197]. In MM, HDACs are considered to function as important oncogenes. Recently, a novel circuitry regulating MM cell growth and survival was identified as miR-29b was proved to specifically target HDAC4. In turn, HDAC4 inhibited miR-29b expression by histone deacetylation that resulted in increased levels of prosurvival targets SP1 and MCL-1 [198] (Table 7).

## 5. Clinical Utility of microRNA Clusters and Families and Epigenetic-Based Therapeutics

A growing number of studies have indicated deregulated expression of miRNAs in cancers and their clinical potential to serve as promising diagnostic, prognostic, and predictive biomarkers as well as novel therapeutic targets. Aberrant DNA hypermethylation of miRNA genes is commonly observed during tumor development and results in downregulation of tumor-suppressive miRNAs. Kunej et al. [69] revealed that miRNAs can be regulated by DNA methylation in only one cancer type, indicating they are cancer type-specific and may be used for better classification of carcinomas with unknown primary tissue of origin. On the other hand, miR-34b was reported to be silenced by DNA methylation in 24 cancer types [96]. Thus, this miRNA could serve as a general cancer biomarker. Importantly, it was shown that digital methylation-specific PCR assay [200] as well as droplet digital PCR [201] may be used to quantify miR-34b/c methylation in serum-circulating DNA of MPM patients, indicating that these approaches could be used for early detection of the disease. Similarly, the feasibility of using miR-34b/c methylation detection in bowel lavage fluid for CRC screening was analyzed. Unfortunately, sensitivity and specificity were not high enough for clinical use [202]. Recently, Ohtsubo et al. [203] analyzed methylation status of 16 tumor-suppressive miRNAs in bile from patients with pancreaticobiliary diseases. They found significantly higher methylation rates of miR-200a/b in patients with PAC compared to benign diseases indicating the clinical utility of this approach for distinguishing between malignant and benign disease. Further, the methylation status of two functional promoters of miR-200b was associated with hormone receptor status in BC patients. While the P1 was hypermethylated in metastatic lymph nodes compared to matched primary tumors, P2 hypermethylation was found in patients with loss of estrogen or progesterone receptor and its hypomethylation was associated with gain of HER2 and androgen receptor expression [121]. These data indicate a potential use of DNA methylation of miRNA promoters for better classification of tumor diseases.

Since the epigenetic status of a tumor may influence its behavior, epigenetic-based therapeutics are tested in several clinical trials with the aim to reprogram cancer cells back to a normal state and overcome chemoresistance, which is one of the major challenges in cancer therapy. Currently, there are six epigenetic drugs approved for clinical use by the FDA, especially in hematologic malignancies [204]. In addition, small molecules inhibiting key enzymes of the epigenetic machinery are widely studied, including DNMT and HDAC inhibitors. Patients with chronic myelomonocytic leukemia (CMML) are frequently treated with hypomethylating agents (HMAs) azacitidine or decitabine. Using these drugs, Berg et al. [205] observed significant upregulation of miR-125a associated with anti-leukemic effects. Importantly, the data were validated in a clinical context, as miR-125a levels increased in CMML patients treated with HMAs, especially in cases showing clinical response to these drugs. Similarly, Chen et al. [168] found that HDACs inhibitors trichostatin A and sodium butyrate significantly upregulated the expression of miR-15a and miR-16 through the increase of histone acetylation in the region of DLEU2/miR-15a/16-1 promoter in LC cells. Subsequently, the expression of BCL-2 decreased, and cell proliferation was reduced. These results indicate that patients with low levels of miR-15a/16 or high levels of HDACs could benefit from HDACs inhibitor-based therapy.

Concerning association with chemoresistance, it was shown that HDAC1 upregulation is a crucial event in drug resistance development in OC. Inhibition of this enzyme by siRNA reduced c-Myc expression, increased miR-34a levels, and sensitized cells to cisplatin-induced apoptosis [111]. Unfortunately, targeting of DNMTs/HDACs is still very unspecific and may lead to severe changes in the whole genome. Since miRNA clusters contain multiple miRNA genes, their targeting may provide better therapeutic outcomes as multiple signaling pathways are affected. However, some miRNA clusters are known to contain miRNAs with dual functions, and activation of such clusters transcription may result in deregulation of several different proteins involved in tumor-suppressive as well as oncogenic signaling pathways. Thus, further research in this area is needed.

Another strategy for epigenetic therapy includes miRNA-based approach. Firstly, miRNA mimics may be used to re-establish the levels of tumor-suppressive miRNAs. Recently, interesting results were published indicating the promising therapeutic potential of miR-489 in triple-negative BC. The authors chemically modified the guide strand of this miRNA by replacing uracil with 5-FU so that tumor-suppressive and DNA damaging components were combined together into a novel therapeutic agent. This drug showed superior effects over miR-489 or 5-FU in inhibition of tumor progression, suggesting its therapeutic efficacy [206]. Inversely, antagomiRs or miRNA sponges are applied to block the function of oncogenic miRNAs. Importantly, binding sites of the sponge are specific to the miRNA seed region, which allows them to block the whole family of related miRNAs [207]. Finally, 22nt long antisense oligonucleotides called miRNA masks are used to compete with miRNAs of interest for 3′-UTR binding sites of target genes. While antagomiRs or miRNA sponges are essential for studying the overall function of particular miRNA, miRNA masks are preferentially used for revealing the specific outcome of regulation of the target gene by the miRNA [208]. Currently, several companies are trying to develop successful miRNA-based cancer therapeutics. In 2013, MiRNA Therapeutics introduced MRX34, a miR-34a mimic, delivered by a liposomal agent Smarticles and tested its potential in patients with various advanced solid tumors. However, this first-in-human clinical trial of a miRNA-based therapy was halted by FDA due to severe immune-mediated toxicities in four patients [209]. Encouraging results were published in the case of Mesomir-1, a TargomiR drug, including miR-16 mimic, bacterially derived minicells, and antibody to EGFR. Recently, phase 1 of clinical trial was completed in MPM or NSCLC patients with acceptable safety profile; however, additional studies are needed [210]. Importantly, a subclass of miRNAs, epi-miRNAs, has been identified. They play an important role in the modulation of epigenome by regulating the expression of key enzymes of epigenetic machinery; thus, they are considered promising therapeutic targets in cancer. Amodio et al. [198] proved that miR-29b targets HDAC4 and highlighted that both molecules are involved in a functional loop. Firstly, silencing of HDAC4 by shRNAs inhibited cell survival and migration and induced apoptosis and autophagy of MM cells due to the downregulation of SP1 and MCL-1, direct targets of miR-29b. Subsequently, hyperacetylation of miR-29b accompanied by elevated levels of this miRNA was observed just as in the case of SAHA (Vorinostat) treatment. Importantly, overexpression of miR-29b potentiated SAHA activity in a murine xenograft model of human MM. These results indicate that miR-29b could serve as a promising epi-therapeutic approach in the treatment of this disease.

During the several last years, advanced methods for studying the role of miRNA clusters and families in tumor development have been established. Using hierarchical cloning method, Wang et al. [211] constructed a synthetic miRNA cluster, which accommodated 18 different miRNA precursors and demonstrated that the maturation and function of individual precursors are independent of their position in the cluster. Further, genome editing methods, such as CRISPR/Cas9, are being introduced in miRNAs research as a powerful tool to delineate the function and regulation of miRNA clusters and families. In 2018, a study by González-Vallinas et al. [212] was published showing that simultaneous overexpression of miRNAs located on chromosome 14q32 by CRISPR, activating technology promoted migration and invasion of lung adenocarcinoma cells similarly to individual miRNA mimics, including miR-323b-3p, miR-487a-3p, and miR-539-5p. These results indicate that using CRISPR-based strategies, we will be able to further elucidate miRNA clusters’ functionality, which will facilitate the development of novel targeted therapies. Last but not least, computational methods, as well as various databases, are being established to complement costly and time-consuming biological experiments. Although clustered and homologous miRNAs are expressed at various levels due to maturation and degradation processes, they are prone to present similar deregulation patterns in particular tumor types [213]. Using multiple types of data to calculate miRNA and disease similarity based on mutual information, adding miRNA family and cluster information to predict human disease-related miRNAs, a new computational method termed FCMDAP, was developed to improve the prediction accuracy of disease-related miRNAs [214]. Finally, EpimiR database collecting regulations between 19 kinds of epigenetic modifications and 617 miRNAs across seven species may be used to search coordinated regulation between miRNAs and epigenetics [215].

## 6. Conclusions

Deregulated expression of miRNAs is a hallmark of a number of solid tumors as well as hematologic malignancies. Recently, the reciprocal regulation between miRNAs expression and epigenetic machinery has been indicated, and crucial epigenetic enzymes, such as DNMTs, HDACs, HATs, or TETs, were found to be strongly enriched among the epi-miRNAs targets indicating the involvement of miRNAs in key cellular processes, including cell differentiation, pluripotency, or chemoresistance. In this review, we have highlighted the association between aberrant DNA methylation, histone methylation or histone acetylation, and altered miRNAs expression. As miRNAs are frequently clustered in the genome, multiple miRNAs may be produced from the same primary transcript, and their expression is affected by the same epigenetic changes. In addition, miRNAs from the same family may be clustered together or they are expressed from different clusters with a close functional relationship. Importantly, aberrant methylation of serum-circulating miRNAs may be detected with modern and high-sensitive methods, thus epigenetically regulated miRNAs could serve as promising diagnostic, prognostic, and predictive biomarkers as well as novel therapeutic targets. Currently, different strategies for epigenetic-based therapies are being developed, including DNMT/HDAC inhibitors or miRNA-based approaches. However, more work needs to be done to improve specificity and reduce the side effects of these molecules. Further, improved systems for miRNA delivery to the target site must be designed. Finally, a combination of computational applications and laboratory-based experimental data will allow the gain of more detailed knowledge of complicated networks of feedback between miRNAs and epigenetic mechanisms, enabling further development of epigenetic anticancer drugs.

## Figures and Tables

**Table 1:** Epigenetically regulated expression of let-7-5p/98-5p family, miR-125-5p family, and miR-99-5p/100-5p family.

DNA Methylation								
**Cancer Type**	**miRNA**	**miRNA Cluster** **(miRBase 22.1)**	**Methylation Status**	**Epigenetic Regulator**	**miRNA Family (miRBase 22.1)**	**Seed Region ^#^**	**Associated Processes/Targets** **Clinical Outcomes**	**Reference**
**GC**	miR-125a	mir-99b~125a (chr19)	HyperMet	-	miR-125a-3p miR-125-5p	CAGGUGA CCCUGAG	Migration, invasion, proliferation, progression	[89]
**CRC**	miR-125a	mir-99b~125a (chr19)	HyperMet	-	miR-125a-3p miR-125-5p	CAGGUGA CCCUGAG	OS	[88]
**LC**	let-7a-3	let-7a-3~let-7b (chr22)	HyperMet	DNMT1/3B	let-7a-3p/98-3p let-7-5p/98-5p	UAUACAA GAGGUAG	Proliferation, cell adhesion	[84]
**OC**	let-7a-3	let-7a-3~let-7b (chr22)	HypoMet	-	let-7a-3p/98-3p let-7-5p/98-5p	UAUACAA GAGGUAG	IGF2, OS	[81]
**BC**	let-7e	mir-99b~125a (chr19)	HyperMet	-	let-7e-3p let-7-5p/98-5p	UAUACGG GAGGUAG	Cell viability, apoptosis, MAPK1, EZH2, OS, subtype	[86]
**Gliomas**	miR-98	let-7f-2~98 (chrX)	HyperMet	-	let-7a-3p/98-3p let-7-5p/98-5p	UAUACAA GAGGUAG	Migration, invasion, SALL4, aggressiveness, OS	[90]
**MM**	miR-125a	mir-99b~125a (chr19)	HyperMet	-	miR-125a-3p miR-125-5p	CAGGUGA CCCUGAG	-	[87]
**ALL**	let-7b	let-7a-3~let-7b (chr22)	HyperMet	-	let-7a-3p/98-3p let-7-5p/98-5p	UAUACAA GAGGUAG	Apoptosis, cell cycle	[83]
**MDS**	let-7a-3	let-7a-3~let-7b (chr22)	HypoMet	-	let-7a-3p/98-3p let-7-5p/98-5p	UAUACAA GAGGUAG	Age, survival	[85]
**Histone Modifications**								
**Cancer Type**	**miRNA**	**miRNA Cluster** **(miRBase 22.1)**	**Histone Modification**	**Epigenetic Regulator**	**miRNA Family** **(miRBase 22.1)**	**Seed Region ^#^**	**Associated Processes/Targets** **Clinical Outcomes**	**Reference**
**BC**	let-7a-3	let-7a-3~let-7b (chr22)	H3K27me3	-	let-7a-3p/98-3p let-7-5p/98-5p	UAUACAA GAGGUAG	-	[94]
	let-7b	let-7a-3~let-7b (chr22)	H3K27me3	-	let-7a-3p/98-3p let-7-5p/98-5p	UAUACAA GAGGUAG	-	[94]
	let-7e	mir-99b~125 (chr19)	H3K4me3	JARID1B	let-7e-3p let-7-5p/98-5p	UAUACGG GAGGUAG	Proliferation, CCND1	[91]
**LC**	miR-99a	mir-99a~let-7c (chr21)	H4R3me2	PRMT5	miR-99-3p miR-99-5p/100-5p	AAGCUCG ACCCGUA	cell growth, FGFR3, metastasis, stage, LNP, OS	[93]
	miR-99b	mir-99b~125a (chr19)	H4R3me2	PRMT5	miR-99-3p miR-99-5p/100-5p	AAGCUCG ACCCGUA	cell growth, FGFR3, metastasis, stage, LNP, OS	[93]
	miR-100	mir-100~let-7a-2 (chr11)	H4R3me2	PRMT5	miR-100-3p miR-99-5p/100-5p	AAGCUUG ACCCGUA	cell growth, FGFR3, metastasis, stage, LNP, OS	[93]
**HCC**	let-7c	mir-99a~let-7c (chr21)	H3K27me3	EZH2	let-7c-3p let-7-5p/98-5p	UGUACAA GAGGUAG	Liver metastasis	[95]

GC—gastric cancer, CRC—colorectal cancer, LC—lung cancer, OC—ovarian cancer, BC—breast cancer, MM—multiple myeloma, ALL—acute lymphoblastic leukemia, MDS—myelodysplastic syndrome, HCC—hepatocellular carcinoma, HyperMet—hypermethylation, HypoMet—hypomethylation, H3K27me3—histone 3 lysine 27 trimethylation, H3K4me3—histone 3 lysine 4 trimethylation, H4R3me2—histone 4 arginine 3 dimethylation, DNMT—DNA methyltransferase, JARID1B—Jumonji/ARID1B, PRMT5—protein arginine methyltransferase 5, EZH2—enhancer of zeste homolog 2, OS—overall survival, IGF2—insulin-like growth factor 2, MAPK1—mitogen-activated protein kinase 1, SALL4—sal-like protein 4, CCND1—cyclin D1, FGFR3—fibroblast growth factor receptor 3, LNP—lymph node positivity; # the bold sequence indicates the mature miRNA used for in vitro/in vivo experiments (-3p or -5p).

**Table 2:** Epigenetically regulated expression of miR-34-5p/449-5p family and miR-34b-5p/449c-5p family.

DNA Methylation								
Cancer Type	miRNA	miRNA Cluster (miRBase 22.1)	Methylation Status	Epigenetic Regulator	miRNA Family (miRBase 22.1)	Seed Region ^#^	Associated Processes/Targets Clinical Outcomes	Reference
**PAC**	miR-34a	-	HyperMet	-	miR-34a-3p miR-34-5p/449-5p	AAUCAGC **GGCAGUG**	Senescence, cell cycle, CDK6	[97,99]
	miR-34b	mir-34b~34c (chr11)	HyperMet	-	miR-34b-3p miR-34b-5p/449c-5p	AAUCACU AGGCAGU	-	[99]
	miR-34c	mir-34b~34c (chr11)	HyperMet	-	miR-34c-3p miR-34-5p/449-5p	AUCACUA GGCAGUG	-	[99]
**PC**	miR-34a	-	Hyper/ HypoMet	-	miR-34a-3p miR-34-5p/449-5p	AAUCAGC **GGCAGUG**	Senescence, cell cycle, CDK6, stage, Gleason score	[97,117]
	miR-34b	mir-34b~34c (chr11)	HypoMet	-	miR-34b-3p miR-34b-5p/449c-5p	AAUCACU AGGCAGU	PSA level	[117]
	miR-34c	mir-34b~34c (chr11)	HypoMet	-	miR-34c-3p miR-34-5p/449-5p	AUCACUA GGCAGUG	PSA level	[117]
**GC**	miR-34b	mir-34b~34c (chr11)	HyperMet	-	miR-34b-3p miR-34b-5p/449c-5p	AAUCACU **AGGCAGU**	Cell growth	[106]
	miR-34c	mir-34b~34c (chr11)	HyperMet	-	miR-34c-3p miR-34-5p/449-5p	AUCACUA GGCAGUG	Cell growth	[106]
**CRC**	miR-34a	-	HyperMet	-	miR-34a-3p miR-34-5p/449-5p	AAUCAGC GGCAGUG	p53 mutation status	[97,99]
	miR-34b	mir-34b~34c (chr11)	HyperMet	-	miR-34b-3p miR-34b-5p/449c-5p	AAUCACU AGGCAGU	-	[99]
	miR-34c	mir-34b~34c (chr11)	HyperMet	-	miR-34c-3p miR-34-5p/449-5p	AUCACUA GGCAGUG	-	[99]
**OC**	miR-34a	-	HyperMet	-	miR-34a-3p miR-34-5p/449-5p	AAUCAGC GGCAGUG	-	[99]
	miR-34b	mir-34b~34c (chr11)	HyperMet	-	miR-34b-3p miR-34b-5p/449c-5p	AAUCACU AGGCAGU	-	[99]
	miR-34c	mir-34b~34c (chr11)	HyperMet	-	miR-34c-3p miR-34-5p/449-5p	AUCACUA GGCAGUG	-	[99]
**BC**	miR-34a	-	HyperMet HypoMet	DNMT1 TET1	miR-34a-3p miR-34-5p/449-5p	AAUCAGC GGCAGUG	-	[97,99,107]
	miR-34b	mir-34b~34c (chr11)	HyperMet	-	miR-34b-3p miR-34b-5p/449c-5p	AAUCACU AGGCAGU	-	[99]
	miR-34c	mir-34b~34c (chr11)	HyperMet	-	miR-34c-3p miR-34-5p/449-5p	AUCACUA GGCAGUG	-	[99]
**CC**	miR-34c	mir-34b~34c (chr11)	HypoMet	-	miR-34c-3p miR-34-5p/449-5p	AUCACUA GGCAGUG	-	[118]
**UCA**	miR-34a	-	HyperMet	-	miR-34a-3p miR-34-5p/449-5p	AAUCAGC GGCAGUG	-	[99]
	miR-34b	mir-34b~34c (chr11)	HyperMet	-	miR-34b-3p miR-34b-5p/449c-5p	AAUCACU AGGCAGU	-	[99]
	miR-34c	mir-34b~34c (chr11)	HyperMet	-	miR-34c-3p miR-34-5p/449-5p	AUCACUA GGCAGUG	-	[99]
**RCC**	miR-34a	-	HyperMet	-	miR-34a-3p miR-34-5p/449-5p	AAUCAGC GGCAGUG	-	[97,99]
	miR-34b	mir-34b~34c (chr11)	HyperMet	-	miR-34b-3p miR-34b-5p/449c-5p	AAUCACU AGGCAGU	-	[99]
	miR-34c	mir-34b~34c (chr11)	HyperMet	-	miR-34c-3p miR-34-5p/449-5p	AUCACUA GGCAGUG	-	[99]
**STS**	miR-34a	-	HyperMet	-	miR-34a-3p miR-34-5p/449-5p	AAUCAGC GGCAGUG	-	[99]
	miR-34b	mir-34b~34c (chr11)	HyperMet	-	miR-34b-3p miR-34b-5p/449c-5p	AAUCACU AGGCAGU	-	[99]
	miR-34c	mir-34b~34c (chr11)	HyperMet	-	miR-34c-3p miR-34-5p/449-5p	AUCACUA GGCAGUG	-	[99]
**ESCC**	miR-34a	-	HyperMet	-	miR-34a-3p miR-34-5p/449-5p	AAUCAGC GGCAGUG	-	[101]
	miR-34b	mir-34b~34c (chr11)	HyperMet	-	miR-34b-3p miR-34b-5p/449c-5p	AAUCACU AGGCAGU	Early stages, ESCC differentiation	[100,101]
	miR-34c	mir-34b~34c (chr11)	HyperMet	-	miR-34c-3p miR-34-5p/449-5p	AUCACUA GGCAGUG	Early stages, ESCC differentiation	[100,101]
**MPM**	miR-34a	-	HyperMet	-	miR-34a-3p miR-34-5p/449-5p	AAUCAGC GGCAGUG	-	[102]
	miR-34b	mir-34b~34c (chr11)	HyperMet	-	miR-34b-3p miR-34b-5p/449c-5p	AAUCACU **AGGCAGU**	Cell cycle, migration, invasion, motility, apoptosis	[102]
	miR-34c	mir-34b~34c (chr11)	HyperMet	-	miR-34c-3p miR-34-5p/449-5p	AUCACUA **GGCAGUG**	Cell cycle, migration, invasion, motility, apoptosis	[102]
**MB**	miR-449a	mir-449c~449a (chr5)	HyperMet	-	miR-34-5p/449-5p	GGCAGUG	WNT group	[108]
**LSCC**	miR-34a	-	HyperMet	-	miR-34a-3p miR-34-5p/449-5p	AAUCAGC GGCAGUG	Smoking, stage, LNP, OS	[104]
**Melanoma**	miR-34a	-	HyperMet	-	miR-34a-3p miR-34-5p/449-5p	AAUCAGC GGCAGUG	-	[97]
**BLC**	miR-34a	-	HyperMet	DNMT3B	miR-34a-3p miR-34-5p/449-5p	AAUCAGC **GGCAGUG**	Migration, invasion, HNF4γ, NOTCH1	[97,105]
**HCC**	miR-34b	mir-34b~34c (chr11)	HyperMet	-	miR-34b-3p miR-34b-5p/449c-5p	AAUCACU AGGCAGU	-	[103]
**LC**	miR-34a	-	HyperMet	-	miR-34a-3p miR-34-5p/449-5p	AAUCAGC GGCAGUG	-	[97]
**NKTCL**	miR-34a	-	HyperMet	-	miR-34a-3p miR-34-5p/449-5p	AAUCAGC GGCAGUG	-	[98]
**NHL**	miR-34a	-	HyperMet	-	miR-34a-3p miR-34-5p/449-5p	AAUCAGC GGCAGUG	-	[98]
**Histone Modifications**								
**Cancer Type**	**miRNA**	**miRNA Cluster** **(miRBase 22.1)**	**Histone Modification**	**Epigenetic Regulator**	**miRNA Family** **(miRBase 22.1)**	**Seed Region ^#^**	**Associated Processes/Targets** **Clinical Outcomes**	**Reference**
**BC**	miR-34a	-	H3Ac	Linc-ROR	miR-34a-3p miR-34-5p/449-5p	AAUCAGC **GGCAGUG**	Gemcitabine-induced autophagy, apoptosis	[112]
	miR-34b	mir-34b~34c (chr11)	H3K27me3	-	miR-34b-3p miR-34b-5p/449c-5p	AAUCACU AGGCAGU	-	[94]
	miR-34c	mir-34b~34c (chr11)	H3K27me3	-	miR-34c-3p miR-34-5p/449-5p	AUCACUA GGCAGUG	-	[94]
**LC**	miR-449a	mir-449c~449a (chr5)	H3K27me3	SUZ12	miR-34-5p/449-5p	**GGCAGUG**	Metastasis, LNP, MAP2K1	[114]
**CHC**	miR-34a	-	H3K27me3	EZH2	miR-34a-3p miR-34-5p/449-5p	AAUCAGC **GGCAGUG**	Proliferation, clonogenicity, tumor growth, NOTCH1/2, JAGGED1	[119]
**GC**	miR-34a	-	HAc	HDAC1	miR-34a-3p miR-34-5p/449-5p	AAUCAGC **GGCAGUG**	Metastasis, CD44, prognosis	[110]
**OC**	miR-34a	-	HAc	HDAC1	miR-34a-3p miR-34-5p/449-5p	AAUCAGC **GGCAGUG**	Proliferation, apoptosis, chemosensitivity	[111]
**OSS**	miR-449a	mir-449c~449a (chr5)	H3K27me3	-	miR-34-5p/449-5p	**GGCAGUG**	Cell cycle, CDK6, CDC25A	[113]
	miR-449b	mir-449c~449a (chr5)	H3K27me3	-	miR-449b-3p miR-34-5p/449-5p	AGCCACA **GGCAGUG**	Cell cycle, CDK6, CDC25A	[113]
**HCC**	miR-449a	mir-449c~449a (chr5)	HAc	HDAC1-3	miR-34-5p/449-5p	**GGCAGUG**	Proliferation, apoptosis, growth, c-MET	[115]

PAC—pancreatic cancer, PC—prostate cancer, GC—gastric cancer, CRC—colorectal cancer, OC—ovarian cancer, BC—breast cancer, CC—cervical cancer, UCA—urothelial carcinoma, RCC—renal cell carcinoma, STS—soft tissue sarcoma, ESCC—esophageal squamous cell carcinoma, MPM—malignant pleural mesothelioma, MB—medulloblastoma, LSCC—laryngeal squamous cell carcinoma, BLC—bladder cancer, HCC—hepatocellular carcinoma, LC—lung cancer, NKTCL—NK/T-cell lymphoma, NHL—Non-Hodgkin lymphoma, CHC—cholangiocarcinoma, OSS—osteosarcoma, HyperMet—hypermethylation, HypoMet—hypomethylation, H3Ac—histone 3 acetylation, H3K27me3—histone 3 lysine 27 trimethylation, HAc—histone acetylation, DNMT—DNA methyltransferase, TET1—ten-eleven translocation methylcytosine dioxygenase 1, EZH2—enhancer of zeste homolog 2, HDAC—histone deacetylase, CDK6—cyclin dependent kinase 6, PSA—prostate-specific antigen, LNP—lymph node positivity, OS—overall survival, HNF4γ—hepatocyte nuclear factor 4 gamma, MAP2K1—dual specificity mitogen-activated protein kinase kinase 1, CDC25A—cell division cycle 25A; # the bold sequence indicates the mature miRNA used for in vitro/in vivo experiments (-3p or -5p).

**Table 3:** Epigenetically regulated expression of miR-141-3p/200a-3p family, miR-200ab-5p family, and miR-200bc-3p/429 family.

DNA Methylation								
Cancer Type	miRNA	miRNA Cluster (miRBase 22.1)	Methylation Status	Epigenetic Regulator	miRNA Family (miRBase 22.1)	Seed Region ^#^	Associated Processes/Targets Clinical Outcomes	Reference
**PAC**	miR-200a	mir-200b~429 (chr1)	HypoMet	-	miR-141-3p/200a-3p miR-200ab-5p	**AACACUG** AUCUUAC	Serum, SIP1 methylation	[124,126]
	miR-200b	mir-200b~429 (chr1)	HypoMet	-	miR-200bc-3p/429 miR-200ab-5p	**AAUACUG** AUCUUAC	Serum, SIP1 methylation	[124,126]
	miR-200c	mir-200c~141 (chr12)	HyperMet	-	miR-200bc-3p/429 miR-200c-5p/550a-3p	**AAUACUG** GUCUUAC	Invasion, WIPF1, OS	[126]
	miR-141	mir-200c~141 (chr12)	HyperMet	-	miR-141-3p/200a-3p miR-141-5p	**AACACUG** AUCUUCC	Invasion, WIPF1, OS	[126]
	miR-429	mir-200b~429 (chr1)	HypoMet	-	miR-200bc-3p/429	AAUACUG	-	[126]
**PC**	miR-200c	mir-200c~141 (chr12)	HyperMet	DNMT1	miR-200bc-3p/429 miR-200c-5p/550a-3p	**AAUACUG** GUCUUAC	Apoptosis, clonogenicity, cell growth, DNMT3A, TET1/3	[134]
	miR-141	mir-200c~141 (chr12)	HyperMet	DNMT1	miR-141-3p/200a-3p miR-141-5p	**AACACUG** AUCUUCC	DNMT3A, TET1/3	[134]
**GC**	miR-200a	mir-200b~429 (chr1)	HyperMet	-	miR-141-3p/200a-3p miR-200ab-5p	AACACUG AUCUUAC	*H. pylori* infection	[133]
	miR-200b	mir-200b~429 (chr1)	HyperMet	-	miR-200bc-3p/429 miR-200ab-5p	AAUACUG AUCUUAC	*H. pylori* infection	[133]
**BLC**	miR-200a	mir-200b~429 (chr1)	HyperMet	-	miR-141-3p/200a-3p miR-200ab-5p	AACACUG AUCUUAC	Invasiveness, differentiation	[122]
	miR-200b	mir-200b~429 (chr1)	HyperMet	-	miR-200bc-3p/429 miR-200ab-5p	**AAUACUG** AUCUUAC	Invasiveness, differentiation, CDDP sensitivity, OS	[122,123]
	miR-200c	mir-200c~141 (chr12)	HyperMet	-	miR-200bc-3p/429 miR-200c-5p/550a-3p	**AAUACUG** GUCUUAC	Invasiveness, differentiation, prognosis of T1 tumors	[122]
**HCC**	miR-200b	mir-200b~429 (chr1)	HyperMet	-	miR-200bc-3p/429 miR-200ab-5p	**AAUACUG** AUCUUAC	Prognosis, BMI1, ZEB1	[125]
**BC**	miR-200b	mir-200b~429 (chr1)	HyperMet HypoMet	DNMT3A/ Kindlin 2/MYC TET1-3	miR-200bc-3p/429 miR-200ab-5p	**AAUACUG** AUCUUAC	Invasion, metastasis, SOX2, CD133, EMT, BC subtype	[121,129,130,131,132]
	miR-200a	mir-200b~429 (chr1)	HyperMet HypoMet	DNMT3A/ FEN1/PCNA TET1-3	miR-141-3p/200a-3p miR-200ab-5p	AACACUG **AUCUUAC**	Proliferation, MET, EGFR	[128,131,132]
**CC**	miR-200b	mir-200b~429 (chr1)	HypoMet	-	miR-200bc-3p/429 miR-200ab-5p	**AAUACUG** AUCUUAC	HIPK3, RBBP6	[118]
**oral**	miR-200a	mir-200b~429 (chr1)	HyperMet	-	miR-141-3p/200a-3p miR-200ab-5p	AACACUG AUCUUAC	OS, smokers/chewers	[127]
	miR-200b	mir-200b~429 (chr1)	HyperMet	-	miR-200bc-3p/429 miR-200ab-5p	AAUACUG AUCUUAC	OS, smokers/chewers	[127]
**MM**	miR-200c	mir-200c~141 (chr12)	HyperMet	-	miR-200bc-3p/429 miR-200c-5p/550a-3p	**AAUACUG** GUCUUAC	Proliferation, migration, colony formation	[87]
**Histone Modifications**								
**Cancer Type**	**miRNA**	**miRNA Cluster** **(miRBase 22.1)**	**Histone Modification**	**Epigenetic Regulator**	**miRNA Family** **(miRBase 22.1)**	**Seed Region ^#^**	**Associated Processes/Targets** **Clinical Outcomes**	**Reference**
**BC**	miR-200a	mir-200b~429 (chr1)	H3K27me3 HAc	EZH2/SUZ12 HDAC2/PELP1	miR-141-3p/200a-3p miR-200ab-5p	**AACACUG** AUCUUAC	CSCs, metastasis, ZEB1/2, OS	[135,140]
	miR-200b	mir-200b~429 (chr1)	H3K27me3	EZH2/SUZ12	miR-200bc-3p/429 miR-200ab-5p	**AAUACUG** AUCUUAC	CSCs	[135]
	miR-429	mir-200b~429 (chr1)	H3K27me3	EZH2/SUZ12	miR-200bc-3p/429	AAUACUG	CSCs	[135]
	miR-141	mir-200c~141 (chr12)	HAc	HDAC2/PELP1	miR-141-3p/200a-3p miR-141-5p	**AACACUG** AUCUUCC	metastasis, ZEB1/2, OS	[140]
**LC**	miR-200a	mir-200b~429 (chr1)	H3K4me3	JARID1B	miR-200bc-3p/429 miR-200ab-5p	**AAUACUG** AUCUUAC	EMT, ZEB1/2	[139]
	miR-200c	mir-200c~141 (chr12)	H3K4me3	JARID1B	miR-200bc-3p/429 miR-200c-5p/550a-3p	**AAUACUG** GUCUUAC	EMT, ZEB1/2	[139]
	miR-200b	mir-200b~429 (chr1)	H3Ac	HDAC1/4	miR-200bc-3p/429 miR-200ab-5p	**AAUACUG** AUCUUAC	Proliferation, apoptosis, cell cycle, chemoresistance, E2F3	[141]
**GC**	miR-200a	mir-200b~429 (chr1)	H3K27me3	EZH2	miR-141-3p/200a-3p miR-200ab-5p	**AACACUG** AUCUUAC	Disease progression	[136]
	miR-200b	mir-200b~429 (chr1)	H3K27me3	EZH2	miR-200bc-3p/429 miR-200ab-5p	**AAUACUG** AUCUUAC	Disease progression	[136]
	miR-429	mir-200b~429 (chr1)	H3K27me3	EZH2	miR-200bc-3p/429	AAUACUG	Disease progression	[136]
**CC**	miR-200b	mir-200b~429 (chr1)	H3K27me3	EZH2/PVT1	miR-200bc-3p/429 miR-200ab-5p	**AAUACUG** AUCUUAC	Proliferation, cell cycle, migration	[137]
**HCC**	miR-200b	mir-200b~429 (chr1)	H3K27me3 H3Ac	EZH2/GIHCG hnRNP U/PCAF/ RNA Pol II/H19	miR-200bc-3p/429 miR-200ab-5p	**AAUACUG** AUCUUAC	Proliferation, migration, liver metastasis, EMT, tumor size, stage, OS	[95,138,143]
	miR-200a	mir-200b~429 (chr1)	H3K27me3 H3Ac	EZH2/GIHCG hnRNP U/PCAF/ RNA Pol II/H19	miR-141-3p/200a-3p miR-200ab-5p	**AACACUG** AUCUUAC	Proliferation, migration, metastasis, EMT, tumor size, stage, OS	[138,143]
	miR-429	mir-200b~429 (chr1)	H3K27me3 H3Ac	EZH2/GIHCG hnRNP U/PCAF/ RNA Pol II/H19	miR-200bc-3p/429	AAUACUG	Proliferation, migration, metastasis, EMT, tumor size, stage, OS	[138,143]
	miR-200c	mir-200c~141 (chr12)	H3Ac	hnRNP U/PCAF/ RNA Pol II/H19	miR-200bc-3p/429 miR-200c-5p/550a-3p	**AAUACUG** GUCUUAC	EMT, metastasis	[143]
	miR-141	mir-200c~141 (chr12)	H3Ac	hnRNP U/PCAF/ RNA Pol II/H19	miR-141-3p/200a-3p miR-141-5p	**AACACUG** AUCUUCC	EMT, metastasis	[143]
**glioma**	miR-200a	mir-200b~429 (chr1)	H3K27me3	EZH2	miR-141-3p/200a-3p miR-200ab-5p	**AACACUG** AUCUUAC	Disease progression	[136]
	miR-200b	mir-200b~429 (chr1)	H3K27me3	EZH2	miR-200bc-3p/429 miR-200ab-5p	**AAUACUG** AUCUUAC	Disease progression	[136]
	miR-429	mir-200b~429 (chr1)	H3K27me3	EZH2	miR-200bc-3p/429	AAUACUG	Disease progression	[136]

PAC—pancreatic cancer, PC—prostate cancer, GC—gastric cancer, BLC—bladder cancer, HCC—hepatocellular carcinoma, BC—breast cancer, CC—cervical cancer, MM—multiple myeloma, LC—lung cancer, HypoMet—hypomethylation, HyperMet—hypermethylation, H3K27me3—histone 3 lysine 27 trimethylation, HAc—histone acetylation, H3K4me3—histone 3 lysine 4 trimethylation, H3Ac—histone 3 acetylation, DNMT—DNA methyltransferase, TET1-3—ten-eleven translocation methylcytosine dioxygenase, FEN1—flap endonuclease 1, PCNA—proliferating cell nuclear antigen, EZH2—enhancer of zeste homolog 2, HDAC—histone deacetylase, PELP1—proline-, glutamic acid- and leucine-rich protein 1, JARID1B—Jumonji/ARID1B, PVT1—plasmacytoma variant translocation 1, hnRNP U—heterogenous nuclear ribonucleoprotein U, PCAF—P300/CBP-associated factor, RNA Pol II—RNA polymerase II, SIP1—Smad-interacting protein 1, WIPF1—WAS/WASL-interacting protein family member 1, OS—overall survival, CDDP—cisplatin, BMI1—polycomb complex protein, ZEB1/2—zinc finger E-box-binding homeobox ½, EMT—epithelial-mesenchymal transition, EGFR—epidermal growth factor receptor, HIPK3—homeodomain interacting protein kinase 3, RBBP6—RB binding protein 6, CSCs—cancer stem cells; # the bold sequence indicates the mature miRNA used for in vitro/in vivo experiments (-3p or -5p).

**Table 4:** Epigenetically regulated expression of mir-17~92a-1 cluster and mir-106a~363 cluster.

DNA Methylation								
Cancer Type	miRNA	miRNA Cluster (miRBase 22.1)	Methylation Status	Epigenetic Regulator	miRNA Family (miRBase 22.1)	Seed Region ^#^	Associated Processes/Targets Clinical Outcomes	Reference
**PAC**	miR-19a	mir-17~92a-1 (chr13)	HyperMet	DNMT1	miR-19-3p miR-19-5p	GUGCAAA **GUUUUGC**	CSCs	[152]
	miR-19b	mir-17~92a-1 (chr13)	HyperMet	DNMT1	miR-19-3p miR-19-5p	GUGCAAA **GUUUUGC**	CSCs	[152]
**GC**	miR-19a	mir-17~92a-1 (chr13)	HypoMet	MeCP2	miR-19-3p miR-19-5p	GUGCAAA **GUUUUGC**	MeCP2, MDR	[145]
	miR-19b	mir-17~92a-1 (chr13) mir-106a~363 (chrX)	HypoMet	MeCP2	miR-19-3p miR-19-5p	GUGCAAA **GUUUUGC**	MeCP2, MDR	[145]
	miR-106a	mir-106a~363 (chrX)	HypoMet	-	miR-106a-3p miR-17-5p/519-3p	UGCAAUG **AAAGUGC**	LNP, stage, diagnosis	[148]
**HCC**	miR-106a	mir-106a~363 (chrX)	HypoMet	-	miR-106a-3p miR-17-5p/519-3p	UGCAAUG **AAAGUGC**	Invasiveness, cell cycle, apoptosis, TP53INP1, CDKN1A	[147]
**glioma**	miR-20a	mir-17~92a-1 (chr13)	HypoMet	DNMT1	miR-20a-3p miR-17-5p/519-3p	CUGCAUU **AAAGUGC**	TMZ-resistance, apoptosis, cell cycle, tumor growth, LRIG1	[146]
**ESCC**	miR-20b	mir-106a~363 (chrX)	HypoMet	-	miR-20b-3p miR-17-5p/519-3p	CUGUAGU **AAAGUGC**	Proliferation, migration, RB1, TP53INP1, LNP, stage, OS	[149]
**melanoma**	miR-18b	mir-106a~363 (chrX)	HyperMet	-	miR-18b-3p miR-18-5p	GCCCUAA **AAGGUGC**	colony formation, apoptosis, tumor growth, EMT, MDM2	[153]
**GB**	miR-18b	mir-106a~363 (chrX)	HyperMet	DNMT1/EZH2/PVT1	miR-18b-3p miR-18-5p	GCCCUAA **AAGGUGC**	Proliferation, HIF1α, prognosis	[154]
**NKTCL**	miR-20b	mir-106a~363 (chrX)	HyperMet	-	miR-20b-3p miR-17-5p/519-3p	CUGUAGU **AAAGUGC**	STAT3	[150]
**Histone Modifications**								
**Cancer Type**	**miRNA**	**miRNA Cluster** **(miRBase 22.1)**	**Histone Modification**	**Epigenetic Regulator**	**miRNA Family** **(miRBase 22.1)**	**Seed Region ^#^**	**Associated Processes/Targets** **Clinical Outcomes**	**Reference**
**ALL**	miR-17	mir-17~92a-1 (chr13)	H3ac H3K4me3	-	miR-17-3p miR-17-5p/519-3p	CUGCAGU **AAAGUGC**	Proliferation, apoptosis, colony formation, MLL-rear.	[155]
	miR-18a	mir-17~92a-1 (chr13)	H3ac H3K4me3	-	miR-18a-3p miR-18-5p	CUGCCCU **AAGGUGC**	Proliferation, apoptosis, colony formation, MLL-rear.	[155]
	miR-19a	mir-17~92a-1 (chr13)	H3ac H3K4me3	-	miR-19-3p miR-19-5p	**GUGCAAA** GUUUUGC	Proliferation, apoptosis, colony formation, MLL-rear.	[155]
	miR-20a	mir-17~92a-1 (chr13)	H3ac H3K4me3	-	miR-20a-3p mir-17-5p/519-3p	CUGCAUU **AAAGUGC**	Proliferation, apoptosis, colony formation, MLL-rear.	[155]
	miR-19b-1	mir-17~92a-1 (chr13)	H3ac H3K4me3	-	miR-19-3p miR-19-5p	**GUGCAAA** GUUUUGC	Proliferation, apoptosis, colony formation, MLL-rear.	[155]
	miR-92a-1	mir-17~92a-1 (chr13)	H3ac H3K4me3	-	miR-25-3p/367-3p miR-92a-1-5p	**AUUGCAC** GGUUGGG	Proliferation, apoptosis, colony formation, MLL-rear.	[155]
**DLBCL**	miR-19a	mir-17~92a-1 (chr13)	HAc	HDAC1/2	miR-19-3p miR-19-5p	**GUGCAAA** GUUUUGC	BARD1 Vorinostat treatment	[157]
	miR-19b	mir-17~92a-1 (chr13) mir-106a~363 (chrX)	HAc	HDAC1/2	miR-19-3p miR-19-5p	**GUGCAAA** GUUUUGC	BARD1 Vorinostat treatment	[157]

PAC—pancreatic cancer, GC—gastric cancer, HCC—hepatocellular carcinoma, ESCC—esophageal squamous cell carcinoma, GB—gallbladder cancer, NKTCL—NK/T-cell lymphoma, ALL—acute lymphoblastic leukemia, DLBCL—diffuse large B-cell lymphoma, HyperMet—hypermethylation, HypoMet—hypomethylation, H3Ac—histone 3 acetylation, H3K4me3—histone 3 lysine 4 trimethylation, DNMT—DNA methyltransferase, MeCP2—methyl CpG-binding protein 2, EZH2—enhancer of zeste homolog 2, PVT1—plasmocytoma variant translocation 1, HDAC—histone deacetylase, CSCs—cancer stem cells, MDR—multidrug resistance, LNP—lymph node positivity, CDKN1A—cyclin dependent kinase inhibitor 1A, TMZ—temozolomide, LRIG1—LRIG1—leucine rich repeats and immunoglobulin like domains 1, RB1—retinoblastoma associated protein 1, OS—overall survival, EMT—epithelial-mesenchymal transition, MDM2—mouse double minute 2 homolog, HIF1α—hypoxia inducible factor 1 α, STAT3—signal transducer and activator of transcription 3, MLL—mixed-lineage leukemia, BARD1—BRCA-1 associated RING domain 1; # the bold sequence indicates the mature miRNA used for in vitro/in vivo experiments (-3p or -5p).

**Table 5:** Epigenetically regulated expression of miR-15-5p/16-5p/195-5p/424-5p/497-5p family.

DNA Methylation								
Cancer Type	miRNA	miRNA Cluster (miRBase 22.1)	Methylation Status	Epigenetic Regulator	miRNA Family (miRBase 22.1)	Seed Region ^#^	Associated Processes/Targets Clinical Outcomes	Reference
**PC**	miR-195	mir-497~195 (chr17)	HyperMet	-	miR-16-2-3p/195-3p miR-15-5p/497-5p	CAAUAUU **AGCAGCA**	Proliferation, migration, EMT	[163]
**GC**	miR-497	mir-497~195 (chr17)	HyperMet	-	miR-497-3p miR-15-5p/497-5p	AAACCAC **AGCAGCA**	Proliferation, invasion, apoptosis, RAF1, stage	[161]
**HCC**	miR-195	mir-497~195 (chr17)	HyperMet	-	miR-16-2-3p/195-3p miR-15-5p/497-5p	CAAUAUU AGCAGCA	-	[162]
	miR-497	mir-497~195 (chr17)	HyperMet	-	miR-497-3p miR-15-5p/497-5p	AAACCAC AGCAGCA	-	[162]
**OC**	miR-424	mir-424~450b (chrX)	HyperMet	-	miR-424-3p miR-15-5p/497-5p	AAAACGU **AGCAGCA**	Proliferation, migration, KIF23	[166]
**BC**	miR-195	mir-497~195 (chr17)	HyperMet	-	miR-16-2-3p/195-3p miR-15-5p/497-5p	CAAUAUU **AGCAGCA**	Proliferation, invasion, RAF1, CCND1	[159]
	miR-497	mir-497~195 (chr17)	HyperMet	-	miR-497-3p miR-15-5p/497-5p	AAACCAC **AGCAGCA**	Proliferation, invasion, RAF1, CCND1, MUC1	[159,160]
**glioma**	miR-424	mir-424~450b (chrX)	HyperMet	-	miR-424-3p miR-15-5p/497-5p	AAAACGU **AGCAGCA**	Invasion, apoptosis, grade, IDH mutation	[164]
CC	miR-424	mir-424~450b (chrX)	HyperMet	-	miR-424-3p miR-15-5p/497-5p	AAAACGU **AGCAGCA**	HIPK3, RBBP6	[118]
EEA	miR-424	mir-424~450b (chrX)	HyperMet	-	miR-424-3p miR-15-5p/497-5p	AAAACGU **AGCAGCA**	CCND1, RICTOR	[165]
AML	miR-15a	mir-15a~16-1 (chr13)	HyperMet	-	miR-15a-3p miR-15-5p/497-5p	AGGCCAU **AGCAGCA**	Prognosis, diagnosis BCL2, ROR1	[158]
	miR-15b	mir-15b~16-2 (chr3)	HyperMet	-	miR-15b-3p miR-15-5p/497-5p	GAAUCAU **AGCAGCA**	Prognosis, diagnosis BCL2, ROR1	[158]
**Histone Modifications**								
**Cancer Type**	**miRNA**	**miRNA Cluster** **(miRBase 22.1)**	**Histone Modification**	**Epigenetic Regulator**	**miRNA Family** **(miRBase 22.1)**	**Seed Region ^#^**	**Associated Processes/Targets** **Clinical Outcomes**	**Reference**
**LC**	miR-15a	mir-15a~16-1 (chr13)	HAc	HDAC3	miR-15a-3p miR-15-5p/497-5p	AGGCCAU **AGCAGCA**	Cell growth, colony formation, BCL2	[168]
	miR-16	mir-15a~16-1 (chr13)	HAc	HDAC3	miR-16-1-3p miR-15-5p/497-5p	CAGUAUU **AGCAGCA**	Cell growth, colony formation, BCL2	[168]
**CC**	miR-195	mir-497~195 (chr17)	H3K27me3	EZH2/PVT1	miR-16-2-3p/195-3p miR-15-5p/497-5p	CAAUAUU **AGCAGCA**	EMT, paclitaxel sensitivity	[170]
**HCC**	miR-195	mir-497~195 (chr17)	HAc	HDAC3/SP1	miR-16-2-3p/195-3p miR-15-5p/497-5p	CAAUAUU AGCAGCA	-	[169]
**CLL**	miR-15a	mir-15a~16-1 (chr13)	H3K4me2	HDACs	miR-15a-3p miR-15-5p/497-5p	AGGCCAU AGCAGCA	Apoptosis, MCL-1	[79]
	miR-16	mir-15a~16-1 (chr13) mir-15b~16-2 (chr3)	H3K4me2	HDACs	miR-16-1-3p miR-16-2-3p/195-3p miR-15-5p/497-5p	CAGUAUU CAAUAUU AGCAGCA	Apoptosis, MCL-1	[79]
**NHL**	miR-15	mir-15a~16-1 (chr13)	HAc	HDAC3	miR-15a-3p miR-15-5p/497-5p	AGGCCAU AGCAGCA	c-MYC expression-associated	[167]
	miR-16	mir-15a~16-1 (chr13)	HAc	HDAC3	miR-16-1-3p miR-15-5p/497-5p	CAGUAUU AGCAGCA	c-MYC expression-associated	[167]

PC—prostate cancer, GC—gastric cancer, HCC—hepatocellular carcinoma, OC—ovarian cancer, BC—breast cancer, CC—cervical cancer, EEA—endometrial endometrioid adenocarcinoma, AML—acute myeloid leukemia, LC—lung cancer, CLL—chronic lymphoblastic leukemia, NHL—Non-Hodgkin lymphoma, HyperMet—hypermethylation, HAc—histone acetylation, H3K27me3—histone 3 lysine 27 trimethylation, HDAC—histone deacetylase, EZH2—enhancer of zeste homolog 2, PVT1—plasmocytoma variant translocation 1, SP1—specificity protein 1, EMT—epithelial-mesenchymal transition, KIF23—kinesin-like protein 23, CCND1—cyclin D1, MUC1—mucin 1, IDH—isocitrate dehydrogenase, HIPK3—homeodomain interacting protein kinase 3, RBBP6—RB binding protein 6, BCL2—B-cell lymphoma 2, ROR1—receptor tyrosine kinase like orphan receptor 1, MCL-1—myeloid cell leukemia 1; # the bold sequence indicates the mature miRNA used for in vitro/in vivo experiments (-3p or -5p).

**Table 6:** Epigenetically regulated expression of miR-23-3p family, mir-23b~24-1 cluster, and mir-23a~24-2 cluster.

DNA Methylation								
Cancer Type	miRNA	miRNA Cluster (miRBase 22.1)	Methylation Status	Epigenetic Regulator	miRNA Family (miRBase 22.1)	Seed Region ^#^	Associated Processes/Targets Clinical Outcomes	Reference
**PC**	miR-23b	mir-23b~24-1 (chr9)	HyperMet	-	miR-23-3p miR-23-5p	**UCACAUU** GGGUUCC	Proliferation, migration, cell cycle, colony formation, EMT, SRC, AKT, OS, RFS	[173]
	miR-27a	mir-23a~24-2 (chr19)	Hyper/ HypoMet	-	miR-27-3p miR-27a-5p	UCACAGU **GGGCUUA**	Cell growth, stage	[178]
	miR-24	mir-23b~24-1 (chr9) mir-23a~24-2 (chr19)	HyperMet	-	miR-24-3p miR-24-5p	**GGCUCAG** GCCUACU	Cell growth, apoptosis, racial difference, AR, IGF1, IGFBP5, ETV1	[182]
**GC**	miR-27b	mir-23b~24-1 (chr9)	HyperMet	-	miR-27-3p miR-27b-5p	**UCACAGU** GAGCUUA	Proliferation, invasion, GSPT1, stage, tumor size	[180]
**HCC**	miR-23a	mir-23a~24-2 (chr19)	HypoMet	-	miR-23-3p miR-23-5p	UCACAUU GGGUUCC	-	[162]
	miR-27a	mir-23a~24-2 (chr19)	HypoMet	-	miR-27-3p miR-27a-5p	UCACAGU GGGCUUA	-	[162]
	miR-23b	mir-23b~24-1 (chr9)	HyperMet	-	miR-23-3p miR-23-5p	**UCACAUU** GGGUUCC	Proliferation, migration	[176]
**BC**	miR-27b	mir-23b~24-1 (chr9)	HyperMet	-	miR-27-3p miR-27b-5p	**UCACAGU** GAGCUUA	Invasion, EMT, tamoxifen resistance, HMGB3	[179]
**CC**	miR-23b	mir-23b~24-1 (chr9)	HyperMet	DNMT1	miR-23-3p miR-23-5p	**UCACAUU** GGGUUCC	Apoptosis, uPA, ZEB1, c-MET, HPV-16 E6, C9orf3	[174,177]
**OSS**	miR-23a	mir-23a~24-2 (chr19)	HyperMet	-	miR-23-3p miR-23-5p	**UCACAUU** GGGUUCC	Proliferation, migration, invasion, RUNX2, CXCL12	[171]
**MM**	miR-23b	mir-23b~24-1 (chr9)	HyperMet	-	miR-23-3p miR-23-5p	**UCACAUU** GGGUUCC	Proliferation, apoptosis, colony formation, SP1	[175]
**WM**	miR-23b	mir-23b~24-1 (chr9)	HyperMet	-	miR-23-3p miR-23-5p	**UCACAUU** GGGUUCC	Proliferation, apoptosis, colony formation, SP1	[175]
**Histone Modifications**								
**Cancer Type**	**miRNA**	**miRNA Cluster** **(miRBase 22.1)**	**Histone Modification**	**Epigenetic Regulator**	**miRNA Family** **(miRBase 22.1)**	**Seed Region ^#^**	**Associated Processes/Targets** **Clinical Outcomes**	**Reference**
**BC**	miR-130a	-	H3K27me3	-	miR-130-3p/454-3p miR-23-3p	AGUGCAA UCACAUU	-	[94]
**RMS**	miR-27a	mir-23a~24-2 (chr19)	HAc	HDAC3/ SMARCA4	miR-27-3p miR-27a-5p	UCACAGU **GGGCUUA**	PAX3:FOXO1 fusion, chemosensitivity, entinostat	[183]
**DLBCL**	miR-27b	mir-23b~24-1 (chr9)	HAc	HDAC6	miR-27-3p miR-27b-5p	**UCACAGU** GAGCUUA	Proliferation, viability, MET, OS	[184]

PC—prostate cancer, GC—gastric cancer, HCC—hepatocellular carcinoma, BC—breast cancer, CC—cervical cancer, OSS—osteosarcoma, MM—multiple myeloma, WM—Waldenström’s macroglobulinemia, RMS—rhabdomyosarcoma, DLBCL—diffuse large B-cell lymphoma, HyperMet—hypermethylation, HypoMet—hypomethylation, H3K27me3—histone 3 lysine 27 trimethylation, HAc—histone acetylation, DNMT—DNA methyltransferase, HDAC—histone deacetylase, SMARCA4—SWI/SNF-related matrix associated actin dependent regulator of chromatin, subfamily a, member 4, EMT—epithelial-mesenchymal transition, OS—overall survival, RFS—recurrence free survival, AR—androgen receptor, IGF1—insulin-like growth factor 1, IGFBP5—insulin-like growth factor binding protein 5, ETV1—ETS variant transcription factor 1, GSPT1—G1 to S phase transition 1, HMGB3—high mobility group box 3, uPA—urokinase-type plasminogen activator, ZEB1—zinc finger E-box-binding homeobox 1, HPV-16—human papillomavirus 16, RUNX2—RUNX family transcription factor 2, CXCL12—C-X-C motif chemokine ligand 12, SP1—specificity protein 1, PAX3—paired box gene 3, FOXO1—forkhead box protein O1; # the bold sequence indicates the mature miRNA used for in vitro/in vivo experiments (-3p or -5p).

**Table 7:** Epigenetically regulated expression of miR-130-3p/301-3p/454-3p family and miR-29-3p family.

DNA Methylation								
Cancer Type	miRNA	miRNA Cluster (miRBase 22.1)	Methylation Status	Epigenetic Regulator	miRNA Family (miRBase 22.1)	Seed Region ^#^	Associated Processes/Targets Clinical Outcomes	Reference
**PC**	miR-130b	mir-301b~130b (chr22)	HyperMet	-	miR-130-3p/454-3p miR-130b-5p	**AGUGCAA** CUCUUUC	DNA-damage response, senescence	[186]
	miR-301b	mir-301b~130b (chr22)	HyperMet	-	miR-130-3p/454-3p miR-301a-5p/301b-5p	**AGUGCAA** CUCUGAC	DNA-damage response, senescence	[186]
**OC**	miR-130b	mir-301b~130b (chr22)	HyperMet	-	miR-130-3p/454-3p miR-130b-5p	**AGUGCAA** CUCUUUC	CSF-1, differentiation, stage, MDR	[185]
**CHS**	miR-454	-	HyperMet	DNMT1/EZH2/HOTAIR	miR-130-3p/454-3p miR-454-5p	**AGUGCAA** CCCUAUC	Apoptosis, autophagy, STAT3, ATG12	[188]
**PAC**	miR-29b	mir-29b-2~29c (chr1)	HyperMet	DNMT1	miR-29-3p miR-29b-2-5p	**AGCACCA** UGGUUUC	Migration, angiogenesis	[194]
**GC**	miR-29b	mir-29b-2~29c (chr1)	HyperMet	DNMT3A	miR-29-3p miR-29b-2-5p	**AGCACCA** UGGUUUC	Proliferation, migration, LASP1, DNMT3A, CDH1, prognosis	[191,192]
	miR-29c	mir-29b-2~29c (chr1)	HyperMet	DNMT3A	miR-29-3p miR-29c-5p	**AGCACCA** GACCGAU	Migration, DNMT3A, CDH1	[191]
**OC**	miR-29b	mir-29b-2~29c (chr1)	HyperMet	DNMT3A/B	miR-29-3p miR-29b-2-5p	**AGCACCA** UGGUUUC	DNMT3A/B	[193]
**GBM**	miR-29b	mir-29b-2~29c (chr1)	HyperMet	DCST1-AS1	miR-29-3p miR-29b-2-5p	**AGCACCA** UGGUUUC	Proliferation, OS	[195]
**BL**	miR-29a	mir-29b-1~29a (chr7)	HyperMet	DNMT3B	miR-29-3p miR-29a-5p	**AGCACCA** CUGAUUU	Cell cycle, apoptosis CDK6, DNMT3B, TCL-1, MCL-1	[190]
	miR-29b	mir-29b-1~29a (chr7) mir-29b-2~29c (chr1)	HyperMet	DNMT3B	miR-29-3p miR-29b-1-5p miR-29b-2-5p	**AGCACCA** CUGGUUU UGGUUUC	Cell cycle, apoptosis CDK6, DNMT3B, TCL-1, MCL-1	[190]
	miR-29c	mir-29b-2~29c (chr1)	HyperMet	-	miR-29-3p miR-29c-5p	**AGCACCA** GACCGAU	Cell cycle, apoptosis CDK6, DNMT3B, TCL-1, MCL-1	[190]
**Histone Modifications**								
**Cancer Type**	**miRNA**	**miRNA Cluster** **(miRBase 22.1)**	**Histone Modification**	**Epigenetic Regulator**	**miRNA Family** **(miRBase 22.1)**	**Seed Region ^#^**	**Associated Processes/Targets** **Clinical Outcomes**	**Reference**
**BC**	miR-130a	-	H3K27me3	-	miR-130-3p/454-3p miR-23-3p	AGUGCAA UCACAUU	-	[94]
**CLL**	miR-29b	mir-29b-1~29a (chr7) mir-29b-2~29c (chr1)	H3K4me2	HDACs	miR-29-3p miR-29b-1-5p miR-29b-2-5p	AGCACCA CUGGUUU UGGUUUC	Apoptosis, MCL-1	[79]
**AML**	miR-29b	mir-29b-1~29a (chr7) mir-29b-2~29c (chr1)	HAc H4R3me2	HDAC1/3 PRMT5	miR-29-3p miR-29b-1-5p miR-29b-2-5p	**AGCACCA** CUGGUUU UGGUUUC	SP1, KIT mutations, FLT3	[196,197]
**MCL**	miR-29a	mir-29b-1~29a (chr7)	H4Ac H3K27me3	HDAC3 PRC2/EZH2	miR-29-3p miR-29a-5p	**AGCACCA** CUGAUUU	Viability, colony formation, IGFR1R, CDK6	[199]
	miR-29b	mir-29b-1~29a (chr7) mir-29b-2~29c (chr1)	H4Ac H3K27me3	HDAC3 PRC2/EZH2	miR-29-3p miR-29b-1-5p miR-29b-2-5p	**AGCACCA** CUGGUUU UGGUUUC	Viability, colony formation, IGFR1R, CDK6	[199]
	miR-29c	mir-29b-2~29c (chr1)	H4Ac H3K27me3	HDAC3 PRC2/EZH2	miR-29-3p miR-29c-5p	**AGCACCA** GACCGAU	Viability, colony formation, IGFR1R, CDK6	[199]
**MM**	miR-29b	mir-29b-1~29a (chr7) mir-29b-2~29c (chr1)	HAc	HDAC4	miR-29-3p miR-29b-1-5p miR-29b-2-5p	**AGCACCA** CUGGUUU UGGUUUC	Cell survival, migration SP1, MCL-1	[198]

PC—prostate cancer, OC—ovarian cancer, CHS—chondrosarcoma, PAC—pancreatic cancer, GC—gastric cancer, GBM—glioblastoma multiforme, BL—Burkitt lymphoma, BC—breast cancer, CLL—chronic lymphoblastic leukemia, AML—acute myeloid leukemia, MCL—mixed-lineage leukemia, MM—multiple myeloma, HyperMet—hypermethylation, H3K27me3—histone 3 lysine 27 trimethylation, H3K4me2—histone 3 lysine 4 dimethylation, HAc—histone acetylation, H4R3me2—histone 4 arginine 3 dimethylation, H4Ac—histone 4 acetylation, DNMT—DNA methyltransferase, EZH2—enhancer of zeste homolog 2, HDAC—histone deacetylase, PRMT5—protein arginine methyltransferase 5, PRC2—polycomb repressing complex 2, CSF-1—colony stimulating factor 1, MDR—multidrug resistance, STAT3—signal transducer and activator of transcription 3, LASP1—LIM and SH3 protein 1, CDH1—E-cadherin 1, OS—overall survival, CDK6—cyclin dependent kinase 6, TCL-1—T-cell leukemia/lymphoma 1, MCL1—myeloid cell leukemia 1, SP1—specificity protein 1, FLT3—fms-like tyrosine kinase 3, IGFR1R—insulin-like growth factor receptor 1; # the bold sequence indicates the mature miRNA used for in vitro/in vivo experiments (-3p or -5p).
